# Recent development of pH‐responsive theranostic nanoplatforms for magnetic resonance imaging‐guided cancer therapy

**DOI:** 10.1002/EXP.20220002

**Published:** 2023-03-30

**Authors:** Xu Li, Renye Yue, Guoqiang Guan, Cheng Zhang, Ying Zhou, Guosheng Song

**Affiliations:** ^1^ State Key Laboratory of Chemo/Biosensing and Chemometrics, College of Chemistry and Chemical Engineering Hunan University Changsha P. R. China

**Keywords:** cancer therapy, MRI‐guided, nanoplatform, pH‐responsive

## Abstract

The acidic characteristic of the tumor site is one of the most well‐known features and provides a series of opportunities for cancer‐specific theranostic strategies. In this regard, pH‐responsive theranostic nanoplatforms that integrate diagnostic and therapeutic capabilities are highly developed. The fluidity of the tumor microenvironment (TME), with its temporal and spatial heterogeneities, makes noninvasive molecular magnetic resonance imaging (MRI) technology very desirable for imaging TME constituents and developing MRI‐guided theranostic nanoplatforms for tumor‐specific treatments. Therefore, various MRI‐based theranostic strategies which employ assorted therapeutic modes have been drawn up for more efficient cancer therapy through the raised local concentration of therapeutic agents in pathological tissues. In this review, we summarize the pH‐responsive mechanisms of organic components (including polymers, biological molecules, and organosilicas) as well as inorganic components (including metal coordination compounds, metal oxides, and metal salts) of theranostic nanoplatforms. Furthermore, we review the designs and applications of pH‐responsive theranostic nanoplatforms for the diagnosis and treatment of cancer. In addition, the challenges and prospects in developing theranostic nanoplatforms with pH‐responsiveness for cancer diagnosis and therapy are discussed.

## Graphical abstract

pH‐responsive theranostic nanoplatforms have provided a great opportunity in the combat with cancer. Herein, we summarize the recent progress in pH‐responsive theranostic nanoplatforms that integrate magnetic resonance imaging‐based diagnostics and a variety of therapeutic modes for tumor detection, imaging, and therapy.

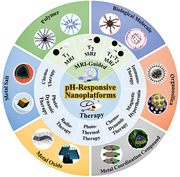

## 1 INTRODUCTION

Nowadays, cancer is one of the most severe illnesses menacing physical health of citizens.^[^ ^1^ ^]^ To fight against cancer, a wide range of treatment methods utilizing the characteristics of tumor microenvironment (TME) have been developed.^[^ ^2^, ^3^, ^4^ ^]^ TME plays a significant part in the initiation and progression of tumors,^[^ ^5^ ^]^ which is also constantly evolving as a consequence of tissue remodeling, metabolic disturbances in the tumor, and alterations in the recruitment of stromal cells.^[^ ^6^ ^]^ On account of the rapid progress of tumor cells and their high demands for metabolism, the TME entails changes and variations in the biochemical conditions within the tissue, such as hypoxia, resulting in the up‐regulated glycolytic metabolism.^[^ ^6^ ^]^ Consequently, the tumor site is more acidic (∼pH 6.8) than healthy tissues on account of the massive accumulation of lactic acid and decreased cellular as well as extracellular pH values thereafter.^[^ ^7^ ^]^ And the pH in early endosomes (pH 5.9–6.2) and late endosomes and lysosomes (pH 5.0–5.5) is even lower.^[^ ^8^ ^]^ The acidic characteristic of the tumor site provides more motivation for tumor growth, invasion, and metastasis,^[^ ^9^ ^]^ but also offers unique chances for pH‐responsive strategies. Compared to other stimuli‐responsive modalities (e.g., laser, ultrasound, and magnetic field), the pH‐responsive strategy would confer more safety because it does not need to be activated by external energy with high power density. Besides, the pH differences between the tumor site and healthy tissues or bloodstream are relatively simple and intrinsic.^[^ ^10^, ^11^ ^]^

In the field of nanomedicine, attention has recently been drawn to the development of theranostic nanoplatforms with both diagnostic and therapeutic abilities, as it provides excellent chances to combat a variety of major diseases.^[^ ^12^, ^13^, ^14^ ^]^ On this account, theranostic nanoplatforms sensitive to the slightly acidic environment are quite advantageous for the detection, imaging as well as therapy of tumors. Owing to passive and active tumor targeting as well as the pH‐sensitivity, theranostic nanoplatforms are competent to deliver therapeutic agents and diagnostic agents specifically to the targeted tumor tissue for concurrent surveillance of pathological process and biological responses to the treatment.^[^ ^15^ ^]^ The main objective of any type of cancer therapy (chemotherapy, radiotherapy, photodynamic therapy (PDT), gene therapy, immunotherapy, hyperthermia, etc.) is to convey the required therapeutic agents specifically to the malignant tumor sites and minimize the side effects to normal tissues. In this respect, the biocompatible and biodegradable nanoplatform, in which cargo release is commonly controlled by pH values and/or other stimuli, is imperative to convey the therapeutic agents specifically to tumor sites, while diminishing any undesirable side effects.^[^ ^16^ ^]^

As one of the most essential components of the theranostic nanoplatform, imaging probes are capable of improving the specificity of the contrast enhancement and providing elaborate characteristics of tumor for clinical diagnosis.^[^ ^12^, ^17^, ^18^, ^19^, ^20^ ^]^ By virtue of the non‐invasive property, magnetic resonance imaging (MRI) technique with non‐limited penetration depth and high spatial resolution is quite competent to provide in vivo tomographic details of pathological tissues in real time.^[^ ^17^, ^21^ ^]^ Consequently, MRI contrast agents (CAs) have turned into one of the most notable diagnostic competitors in fabricating theranostic nanoplatforms.^[^ ^15^, ^22^ ^]^ The selective delivery of CAs such as superparamagnetic iron oxide nanoparticles, Mn(II) complexes, and Gd(III) complexes by tailor‐made nanoplatforms, including block copolymers,^[^ ^23^, ^24^, ^25^, ^26^ ^]^ liposomes,^[^ ^27^ ^]^ graphene oxide,^[^ ^28^, ^29^ ^]^ hydrogels,^[^ ^30^ ^]^ to the targeted tumor site is critical in endowing the theranostic nanosystem with ultrasensitive MRI contrast ability.

This review summarizes the recent progress in pH‐responsive theranostic nanoplatforms that integrate MRI‐based diagnostics and a variety of therapeutic modes for tumor detection, imaging, and therapy. First, we outline the design of pH‐responsive nanoplatforms, which consists of organics‐based and inorganics‐based pH‐responsive nanoplatforms. Second, MRI contrast agents applied in pH‐responsive theranostic nanoplatforms are summarized, such as T_1_‐weighted (T_1_) MRI CAs, T_2_‐weighted (T_2_) MRI CAs, and T_1_‐T_2_ dual modal MRI CAs. Furthermore, a variety of MRI‐guided therapeutic modes, including MRI‐guided single therapeutic mode and MRI‐guided combined therapeutic modes, produced by pH‐responsive theranostic nanoplatforms are discussed. The final section describes the challenges and prospects in developing theranostic nanoplatforms with pH‐responsiveness for cancer diagnosis and therapy.

## 2 THE DESIGN OF PH‐RESPONSIVE NANOPLATFORMS

### 2.1 Polymer‐based pH‐responsive nanoplatforms

Polymer‐based nanoparticles (NPs) have drawn much attention from researchers because of their inherent advantages such as superb biocompatibility, long‐term stability, negligible toxicity, and easy conjugation with functional components. Moreover, some polymers containing stimuli‐responsive functional groups can particularly react to pathological “triggers” connected with environmental characteristics like pH.

**SCHEME 1:**
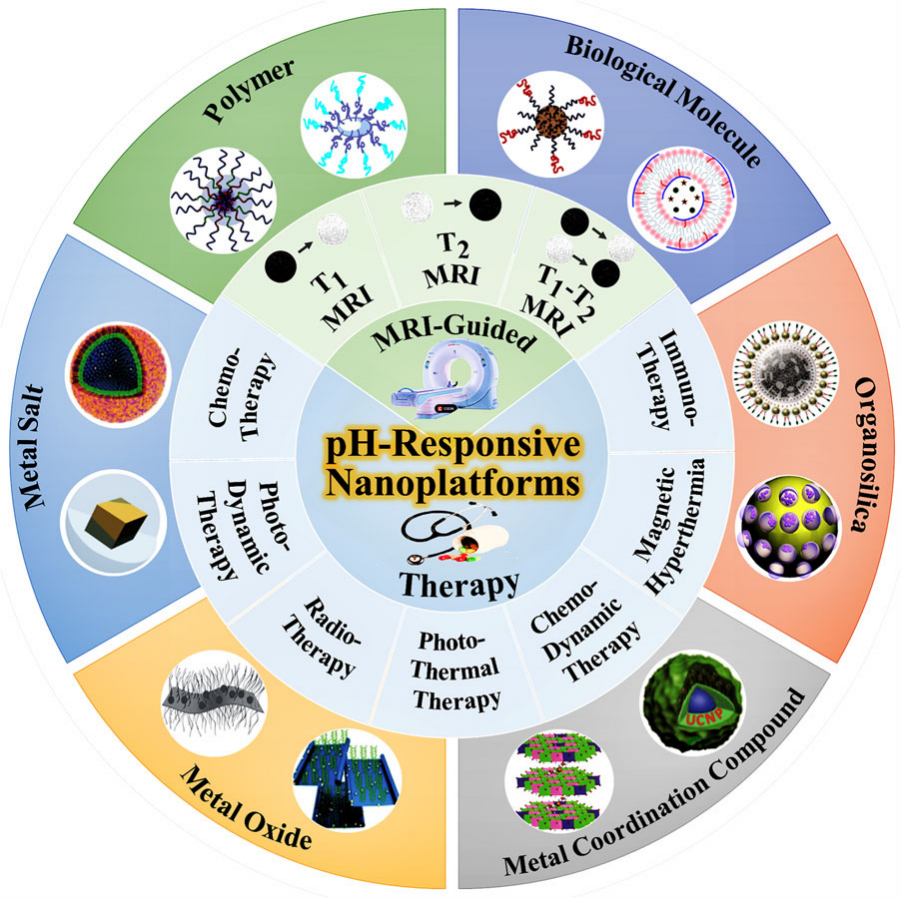
Schematic illustration of pH‐responsive theranostic nanoplatforms. Various therapeutic modes for cancer under magnetic resonance imaging guidance (inner ring). pH‐responsive nanoplatforms based on various nanomaterials (outer ring). Reproduced with permission.^[^ ^23^ ^]^ Copyright 2013, Elsevier Ltd.; Reproduced with permission.^[^ ^24^ ^]^ Copyright 2016, American Chemical Society; Reproduced with permission.^[^ ^58^ ^]^ Copyright 2017, Elsevier Ltd.; Reproduced with permission.^[^ ^27^ ^]^ Copyright 2013, Elsevier B.V.; Reproduced with permission.^[^ ^65^ ^]^ Copyright 2016, American Chemical Society; Reproduced with permission.^[^ ^67^ ^]^ Copyright 2015, Elsevier Ltd.; Reproduced with permission.^[^ ^71^ ^]^ Copyright 2019, Wiley‐VCH Verlag GmbH & Co. KGaA; Reproduced with permission.^[^ ^83^ ^]^ Copyright 2017, WILEY‐VCH Verlag GmbH & Co. KGaA; Reproduced with permission.^[^ ^97^ ^]^ Copyright 2019, Wiley‐VCH Verlag GmbH & Co. KGaA; Reproduced with permission.^[^ ^91^ ^]^ Copyright 2014, WILEY‐VCH Verlag GmbH & Co. KGaA; Reproduced with permission.^[^ ^103^ ^]^ Copyright 2021, American Chemical Society; Reproduced with permission.^[^ ^102^ ^]^ Copyright 2019, WILEY‐VCH Verlag GmbH & Co. KGaA.

**FIGURE 1:**
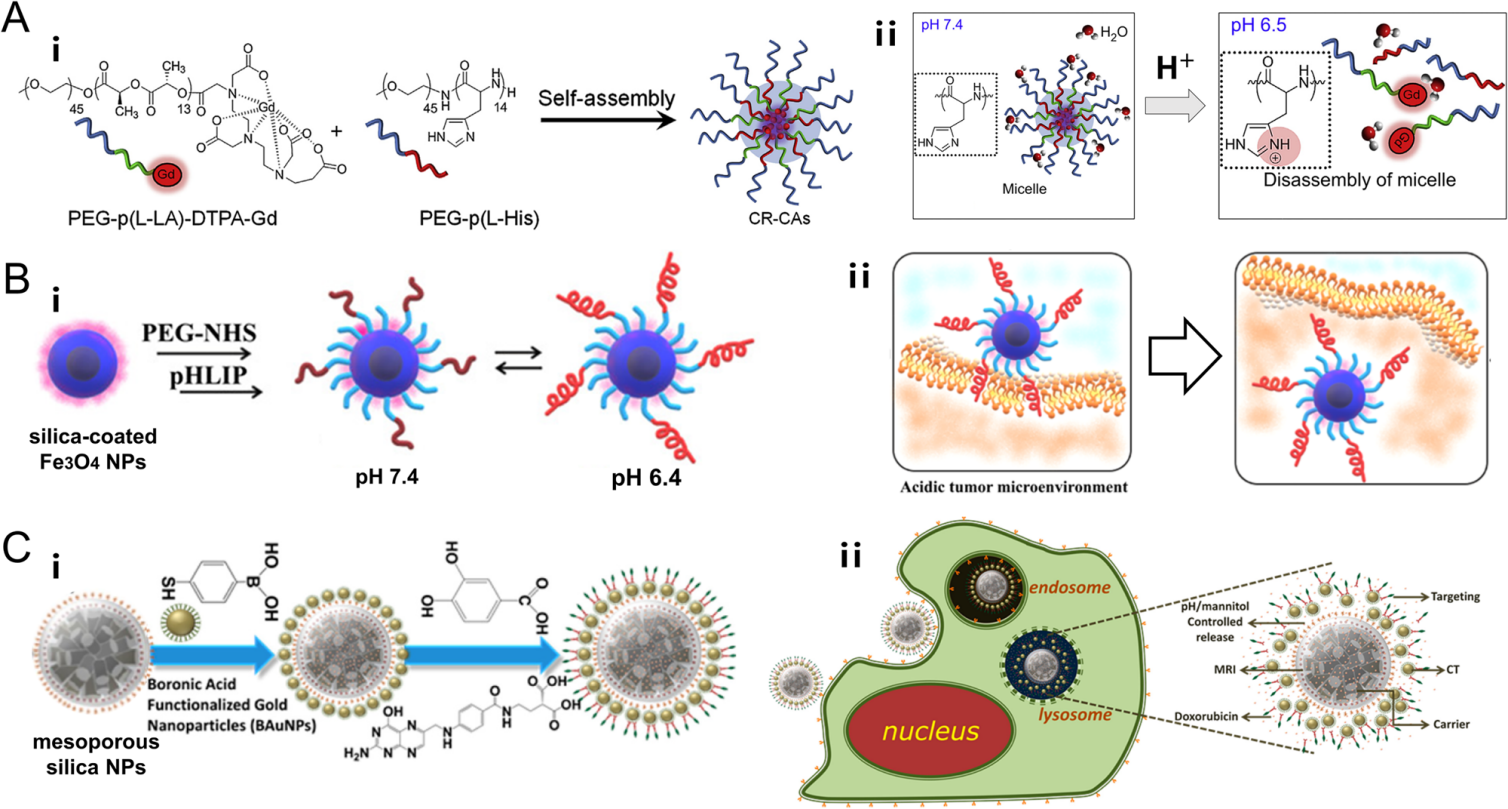
pH‐responsive nanoplatforms based on polymer, biological molecule, and organosilica respectively. (A) Polymer‐based nanoplatform. Schematic representation of the preparation of the cancer‐recognizable magnetic resonance imaging contrast agents (CR‐CAs) and pH‐dependent structural transformation in CR‐CAs. Inset: Chemical structural representation of the protonation of imidazole groups in poly(ethylene glycol)‐*b*‐poly(l‐histidine) (PEG‐p(l‐His)) at acidic pH. Reproduced with permission.^[^ ^23^ ^]^ Copyright 2013, Elsevier Ltd. (B) Biological molecule‐based nanoplatform. Schematic representation of the preparation of pH low insertion peptide (pHLIP)‐modified Fe_3_O_4_ NPs and pH‐dependent structural transformation of pHLIP. Reproduced with permission.^[^ ^59^ ^]^ Copyright 2021, American Chemical Society. (C) Organosilica‐based nanoplatform. Schematic representation of the preparation of gold NPs‐conjugated mesoporous silica NPs and pH‐controlled release. Reproduced with permission.^[^ ^65^ ^]^ Copyright 2016, American Chemical Society.

The pH‐responsive block copolymers comprising ionizable functional groups can provide or accept protons in responding to the environmental pH fluctuation. In a slightly acidic environment, the amino groups can accept protons and be ionized, consequently resulting in an insoluble–soluble transition from hydrophobicity to hydrophilicity. For instance, the pH‐responsive polymeric micelles (CR‐CAs) made from amphiphilic block copolymers were self‐assembled, which consist of poly(ethylene glycol)‐b‐poly(l‐histidine) (PEG‐p(l‐His)) and PEG‐*b*‐poly(l‐lactic acid)‐diethylenetriaminopentaacetic acid dianhydride‐gadolinium chelate. And they would be destabilized as a result of the protonation of amino groups in p(l‐His) blocks under acidic conditions of tumor, leading to their break apart into protonated water‐soluble polymers (Figure 1A).^[^ ^23^ ^]^ In addition, many other block copolymers containing pH‐responsive tertiary amino groups have also been applied to encapsulate hydrophobic contrast agents or drugs for possible acid‐targeting delivery.^[^ ^24^, ^31^ ^]^ For example, Chen et al. employed poly(ethylene glycol)‐ poly(2‐(hexamethyleneimino)ethyl methacrylate) to encapsulate gadolinium metallofullerene‐polypyrrole nanoparticle (denoted as PEG‐GMF‐PPy NP) for pH‐activatable and tumor‐specific imaging.^[^ ^31^ ^]^ Besides, poly(β‐amino ester)s were reported to present weakly basic character due to their tertiary amine groups, which leads to being water‐soluble at a lower pH and insoluble in water at a neutral pH.^[^ ^32^ ^]^ Thus, some researchers have fabricated a great deal of pH‐responsive polymeric micelles by introducing poly(β‐amino ester) block to form pH‐responsive probes.^[^ ^33^, ^34^ ^]^

pH‐sensitive hydrogels comprising ionizable functional groups can respond to alterations in the surrounding environment and unload cargo through pH‐responsive release mechanisms.^[^ ^35^ ^]^ Yang and co‐workers fabricated a PEG‐based hydrogel containing imidazole functional groups to endow the nanoplatform with pH‐responsiveness to tumor acidity. Therefore, the amine functional groups in imidazole moieties are mainly protonated in an acidic environment of tumor, leading to an enhanced water absorption into the hydrogels and rapid release of loaded cargo.^[^ ^30^ ^]^ Lin and co‐workers constructed poly(acrylic acid) (PAA) modified lanthanide–doped GdVO_4_ nanoparticles by filling PAA hydrogel into GdVO_4_ hollow spheres, which exhibited drug‐loading and pH‐dependent drug releasing characteristics.^[^ ^36^ ^]^

As part of the polymer family, poly(acrylic acid) (PAA) which is biodegradable has been considered as a charming candidate for drug delivery as a result of its diminished toxicity, antigenicity, and immunogenicity.^[^ ^37^, ^38^ ^]^ Moreover, the large number of carboxyl functional groups of PAA not only makes the nanoparticles hydrophilic, but can efficiently load some drugs such as doxorubicin (DOX),^[^ ^39^ ^]^ cisplatin,^[^ ^40^ ^]^ through electrostatic interaction, which mainly exists between the charged active functional groups. And the release of drugs from PAA would be triggered in an acidic environment due to the destruction of electrostatic interaction. In addition, polyethylenimine with an abundance of amino groups was coated onto hydroxylated mesoporous nanosilica via electrostatic interactions as well as hydrogen bonds and served as pH‐sensitive gatekeepers for the control of drug release since both electrostatic interactions and hydrogen bonds are sensitive to protons.^[^ ^41^ ^]^

In addition, some other kinds of polymers were employed to conjugate with anticancer drugs through pH‐responsive hydrazone bonds, which could be quickly cleaved to realize controlled drug release in acidic tumor sites. For example, anticancer drug doxorubicin could simply conjugate with hydrazine‐functionalized poly(ethylene glycol) (SH‐PEG) via pH‐sensitive hydrazone bonds.^[^ ^42^ ^]^ Sun et al. linked such conjugation (SH‐PEG‐DOX) onto gold nanocrystals to construct pH‐responsive nanoplatform (denoted as UCNPs@Au‐DOX). And the pH‐responsive hydrazone linkages would be quickly cleaved to achieve targeted drug release in the acidic environment of tumor.^[^ ^43^ ^]^ Similarly, some other researchers have applied hydrazine modified salep,^[^ ^44^ ^]^ hydrazide tailored poly (methyl methacrylate),^[^ ^45^ ^]^ acylhydrazine modified poly (amidoamine) dendrimers^[^ ^46^ ^]^ and block copolymer^[^ ^47^ ^]^ to conjugate with DOX via pH‐responsive hydrazone bonds for targeted drug release.

### 2.2 Biological molecule‐based pH‐responsive nanoplatforms

Chitosan is a natural biodegradable and biocompatible polysaccharide based on chitin and has been widely employed for multiple biomedical applications, namely drug delivery, tissue engineering, and wound healing.^[^ ^48^, ^49^ ^]^ Recently, chitosan has been reported to possess excellent drug loading property as well as improved pH‐sensitive drug release ability.^[^ ^48^ ^]^ In this case, many researchers have reported the synthesis of chitosan‐functionalized nanoplatforms for drug/contrast agents loading and pH‐responsive release.^[^ ^50^, ^51^, ^52^, ^53^ ^]^ In addition, another kind of polysaccharide, carboxymethyl dextran (CMD), which exhibits good biocompatibility and biodegradability, could be used as non‐toxic ingredients in formulations and reagents for binding other components. For example, Zhang et al. chemically modified CMD on the surface of porous manganese phosphate nanoparticles via pH‐sensitive boronate esters (denoted as PMP‐CMD NP), which would be cleaved under acidic conditions of tumor, leading to the dissociation of CMD from the surface.^[^ ^54^ ^]^

pH low insertion peptide (pHLIP) is comprised of 36 amino acids and has exhibited effectiveness and selectivity to target the acidic TME. By means of the protonation of aspartic acid residues in an acidic environment, pHLIP can achieve a conformational transition to develop the α‐helix insertion mode, which is unidirectionally inserted into the membrane with C‐terminal inside.^[^ ^55^, ^56^ ^]^ Consequently, many researchers have applied pHLIP as the pH‐responsive ligands to enhance the accumulation of magnetic nanoparticles within tumor sites, which showed great biomedical potential to target acidic TME for tumor detection and diagnosis through MRI (Figure 1B).^[^ ^57^, ^58^, ^59^ ^]^

Except for pHLIP, some other peptides are also sensitive to acidic TME, generating targeting activity. For instance, Kim and co‐workers reported on a lower pH‐triggered drug‐eluting nanocomposite (denoted as pH‐DEN) consisting of pH‐sensitive synthetic peptides with lipid tails as the gatekeeper. In an acidic environment, the synthetic peptides were destabilized due to ionization of imidazole functional groups and transformed into a charged water‐soluble form, thus triggering the controllable drug release from nanoparticles.^[^ ^60^ ^]^ Lee et al. conjugated 2,3‐dimethylmaleic acid (DMA) with poly(l‐lysine) via the dehydration condensation of carboxyl groups with amino groups and realized surface charge switching for the improvement of tumor recognition efficiency with the dissociation of DMA under acidic TME.^[^ ^61^ ^]^ Moreover, Zheng and co‐workers designed one kind of cell penetrating peptides (CPPs), H_7_K(R_2_)_2_, which can react to the slightly acidic environment of the tumor site. More specifically, this pH‐sensitive peptide would generate CPP characteristics under acidic conditions of tumor and non‐CPP characteristics in a neutral environment of healthy tissues.^[^ ^62^ ^]^

Liposomes have drawn much attention as pH‐responsive platforms owing to their size control properties, biocompatibility, and drug‐loading capacity.^[^ ^63^ ^]^ For instance, nanohybrid liposomes covered by amphiphilic hyaluronic acid–ceramide were developed to specifically deliver anticancer drugs into tumor sites. Drug release from the fabricated nanohybrid liposome could be enhanced at lower pH versus physiological pH.^[^ ^27^ ^]^ Nguyen and co‐workers reported on the liposome‐based polymer‐caged nanobins acting as a nanoscale delivery system, which allowed for a high dose encapsulation of anticancer drugs through an ion‐gradient‐mediated drug‐loading approach. And the nanoplatform exhibited pH‐dependent drug releasing behavior in the acidic environment of tumor.^[^ ^64^ ^]^

### 2.3 Organosilica‐based pH‐responsive nanoplatforms

Although silica exhibits no pH‐responsive property, the organosilica nanoparticles modified with a range of organics could conjugate with other components through pH‐responsive chemical bonds, which would be cleaved under acidic conditions. Among them, boronate ester bond can be formed via the condensation of polyalcohol and boronic acid for the connection of silica nanoparticles with other components. For example, Chou et al. tethered polyalcohol saccharide‐derivative silane on the outside of mesoporous silica nanoparticles to connect with boronic acid modified gold nanoparticles through boronate ester bonds, which can be hydrolyzed under acidic conditions of the tumor and realize pH‐controlled release (Figure 1C).^[^ ^65^ ^]^

Another kind of pH‐responsive chemical bond for the connection of organosilica nanoparticles with other components is imine bond, which can be formed by reacting aldehydes or ketones with amine derivatives. By making use of imine bonds, Zheng and co‐workers sealed hollow mesoporous organosilica nanoparticles with the enzyme catalyst glucose oxidase (GOD). And the blocking GOD gatekeeper could be eliminated through pH‐induced hydrolyzation of imine bonds.^[^ ^66^ ^]^

Additionally, acetals are formed through the condensation of aldehydes and alcohols and can be hydrolyzed in an acidic environment. Based on this characteristic, Lu and co‐workers constructed a system (denoted as MEMSN) by modifying acetals on the outside of mesoporous silica, and then conjugating with ultrasmall lanthanide–doped upconverting nanoparticles as the gate keepers. On account of the hydrolyzation reactions of acetals, the loaded anticancer drug DOX achieved pH‐responsive burst release under acidic conditions of tumor.^[^ ^67^ ^]^

### 2.4 Metal coordination compound nanoparticles‐based pH‐responsive nanoplatforms

The coordination compounds have been regarded as a promising candidate to construct theranostic nanoplatforms as a result of the appealing advantages such as the comparatively stable structure, componential tunability, good biodegradability as well as facile surface modification.^[^ ^68^, ^69^, ^70^ ^]^ Moreover, for some of coordination compounds, the coordinated bonds between metal ions and organic ligands are pH‐sensitive and can be broken up under acidic conditions of the tumor, thus resulting in the disintegration of coordination complexes.

**FIGURE 2:**
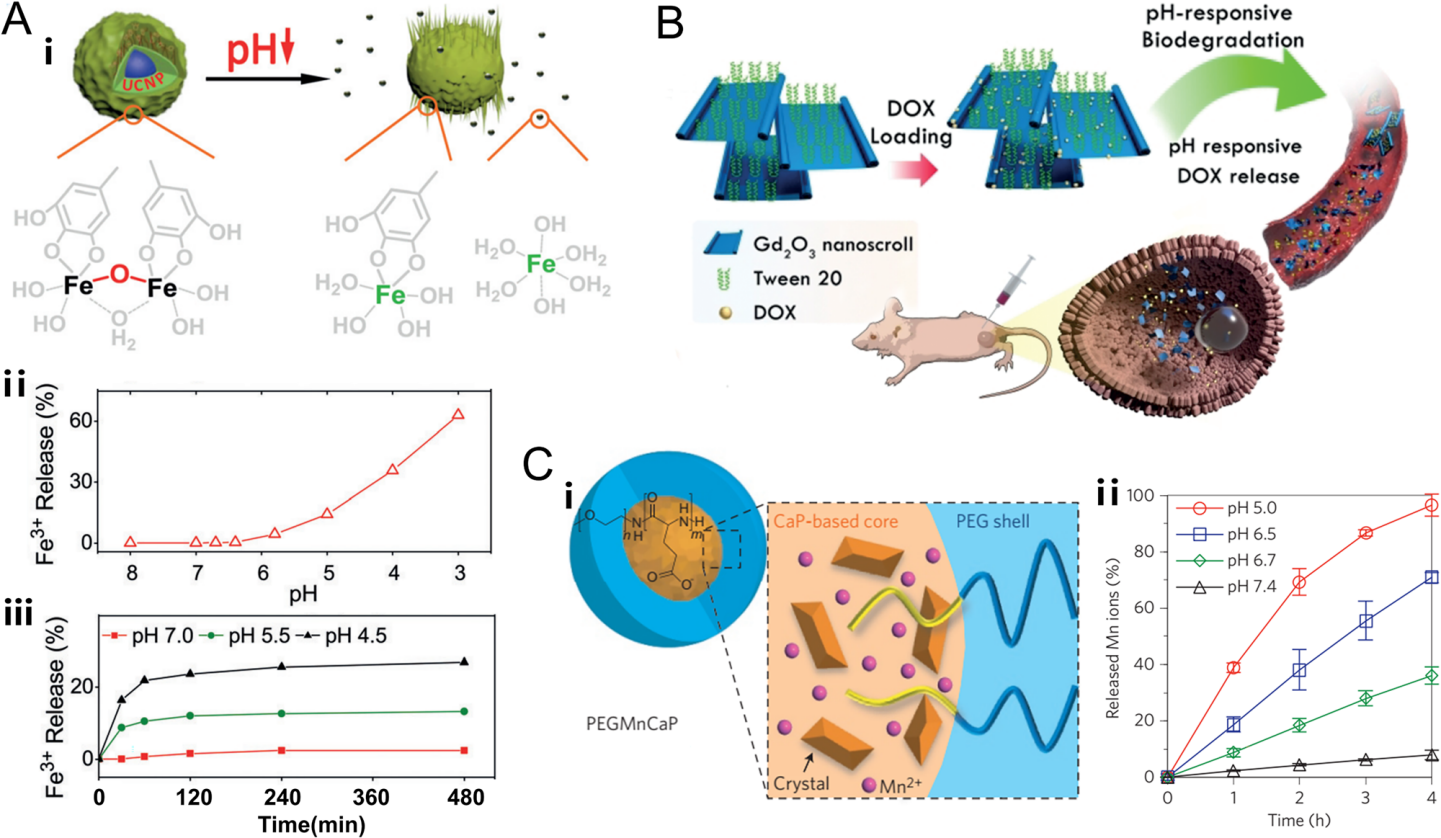
pH‐responsive nanoplatforms based on metal coordination compound, metal oxide, and metal salt respectively. (A) Metal coordination compound nanoparticles‐based nanoplatform. Schematic representation of pH‐dependent disintegration of the coordinatively unsaturated shell (Fe‐GA) and pH‐dependent release of Fe^3+^ at different pH values. Reproduced with permission.^[^ ^71^ ^]^ Copyright 2019, Wiley‐VCH Verlag GmbH & Co. KGaA. (B) Metal oxide nanoparticles‐based nanoplatform. Schematic illustration of the composition of ultrathin Gd_2_O_3_ nanoscrolls and their pH‐responsive biodegradation as well as DOX release. Reproduced with permission.^[^ ^97^ ^]^ Copyright 2019, Wiley‐VCH Verlag GmbH & Co. KGaA. (C) Metal salt nanoparticles‐based nanoplatform. Schematic illustration of the hybrid structure of PEGMnCaP and release profiles of Mn ions under physiological conditions at different pH. Reproduced with permission.^[^ ^106^ ^]^ Copyright 2016, Macmillan Publishers Limited.

Plentiful phenolic hydroxyl functional groups of gallic acid (GA) could strongly coordinate with Fe^3+^ to construct a Fe‐GA network structure, which is quite stable and barely releases ferric ions in the TME.^[^ ^71^ ^]^ To deal with this problem, Gao et al. tuned the stability of the Fe‐GA coordination compound through the adjustment of GA ligand number in which ferric ion is concurrently coordinated with GA and HO^−^ /H_2_O (denoted as UCNP@GA‐Fe). The introduction of such coordinated structure leads to the easier release of ferric ions in the slightly acidic environment (Figure 2A).^[^ ^71^ ^]^ Recently, nanoscale metal–organic‐frameworks (MOFs) have exhibited great potential as promising nanoplatforms for the delivery of therapeutic agents and imaging agents.^[^ ^72^ ^]^ For instance, the MIL family which is fabricated from nontoxic and biodegradable porous ferric ion carboxylate materials with different compositions and structures has demonstrated large capacity for drug loading and pH‐responsive release.^[^ ^73^, ^74^, ^75^ ^]^ Therefore, some researchers employed MIL‐100 which could decompose in an acidic environment to construct pH‐responsive cargo release system.^[^ ^76^, ^77^ ^]^ For example, Chen et al. developed the core–shell MOFs (denoted as CS‐MOFs) and the MIL‐100 outer shell could achieve pH‐dependent delivery of Artesunate.^[^ ^77^ ^]^ Additionally, some other ferric ion‐based coordination complexes such as catechol‐Fe^3+^ coordinated micelles,^[^ ^78^ ^]^ Fe‐polydopamine (PDA) coordinated nanoparticles,^[^ ^79^ ^]^ FeOOH clusters,^[^ ^80^ ^]^ are similarly employed to endow the nanoplatform with pH‐responsive characteristic.

As a 2D nanomaterial, layered double hydroxides (LDHs) possess the positively charged host layers similar to brucite structures consisting of edge‐sharing octahedral units, in which every metal ion is simultaneously coordinated with six hydroxyl groups.^[^ ^81^ ^]^ The protonation of hydroxyl groups surrounding metal cations makes LDHs sensitive to weakly acidic environments. Besides, LDHs could be easily doped with diverse metal cations such as Mn^2+^ in the brucite‐like layers.^[^ ^82^ ^]^ Inspired by these advantages, researchers developed Mn‐based LDHs nanomaterials as enhanced MRI contrast agents with sensitive pH response.^[^ ^83^, ^84^ ^]^ In addition, Zhu et al. reported on the methotrexate–Mn^2+^ (MTX‐Mn) based nanoscale coordination polymers (denoted as MTX‐Mn@PEG), which were proved to be pH‐sensitive and could be decomposed into methotrexate and Mn^2+^ in the intracellular acidic environment of tumor.^[^ ^85^ ^]^

As a member of MOFs family, Zeolitic imidazolate framework‐8 (ZIF‐8) is composed of Zn^2+^ cations and 2‐methyl imidazolate linkers. The coordination interactions between Zn^2+^ ions and imidazolate ions can be affected under acidic conditions because of the effects of protonation, which causes the degradation of ZIF‐8.^[^ ^86^ ^]^ Therefore, it has shown advantageous characteristics of pH‐responsiveness in the slightly acidic TME while staying stable in normal physiological environments and has been employed as the drug storage and delivery vehicle for tumor‐targeted drug release.^[^ ^87^, ^88^ ^]^

### 2.5 Metal oxide nanoparticles‐based pH‐responsive nanoplatforms

Manganese oxide nanomaterials developed in recent years exhibit controllable physical and chemical properties, negligible toxicity, and good T_1_ contrast effects.^[^ ^89^ ^]^ As a TME‐responsive biodegradable agent, manganese oxide nanomaterials have aroused considerable interest in tumor‐targeted theranostics.^[^ ^90^, ^91^ ^]^ Some recent researches revealed that manganese oxide materials have TME‐sensitive and TME‐modulating features, which could be dissolved into Mn^2+^ ions under acidic conditions of the TME.^[^ ^92^ ^]^ For example, Huang et al. coated MnO_2_ onto gold nanorods to construct pH‐responsive theranostic nanoplatform.^[^ ^93^ ^]^ In addition, ultrasmall MnO_2_ nanoparticles were also employed as gate keepers to seal mesoporous silica NPs (denoted as USMO@MSN) for pH‐responsive cargo release.^[^ ^94^ ^]^ Moreover, some other researchers have applied manganese oxide nanomaterials such as manganese oxide nanoparticles,^[^ ^95^ ^]^ honeycomb manganese oxide nanospheres,^[^ ^96^ ^]^ Two‐Dimensional MnO_2_ Nanosheets^[^ ^91^ ^]^ to construct pH‐responsive degradable nanocarriers.

Apart from manganese oxide, some other metallic oxide nanomaterials were also reported to be pH‐sensitive and were employed to endow the nanoplatform with pH‐responsive property. For instance, by making use of gadolinium oxide nanomaterials, Du et al. synthesized pH‐responsive Gd_2_O_3_ nanoscrolls, which could biodegrade upon encountering the TME and realize targeted delivery of loaded drugs (Figure 2B).^[^ ^97^ ^]^ Wang et al. employed the hollow Gd_2_O_3_ nanospheres to efficiently load tumor antigens for pH‐dependent release.^[^ ^98^ ^]^ In addition, Hou et al. reported on the pH‐sensitive Fe_5_C_2_@Fe_3_O_4_ nanoparticles in which Fe_3_O_4_ shell can discharge ferrous ions effectively for the catalysis of the Fenton reaction and T_1_ MRI in acidic environments.^[^ ^99^ ^]^ Zhang et al. applied ZnO as the gatekeepers to effectively encapsulate the drugs in the mesopores of mesoporous silica NPs till it is disintegrated under acidic conditions of the tumor to achieve on‐demand drug release.^[^ ^12^ ^]^

### 2.6 Metal salt nanoparticles‐based pH‐responsive nanoplatforms

Calcium carbonate (CaCO_3_) and manganese carbonate (MnCO_3_) offer attractive characteristics such as being highly sensitive to acidic environments on account of their intrinsic nature of comprising Ca^2+^/Mn^2+^ and CO_3_^2−^. Therefore, some researchers took advantage of the pH‐sensitivity of calcium carbonate to construct pH‐responsive theranostic nanoplatform.^[^ ^100^, ^101^, ^102^ ^]^ Meanwhile, other researchers employed manganese carbonate to endow the nanomaterials with pH‐activatable property within the tumor site.^[^ ^103^, ^104^ ^]^

By virtue of their promise in offering pH‐responsive diagnostic functions and therapeutic benefits concurrently, biodegradable and biocompatible metal phosphate nanomaterials have been demonstrated to possess huge potential in tumor theranostic applications.^[^ ^54^ ^]^ Thus, Zhang et al. developed tumor acidity‐activatable nanoplatform based on manganese phosphate with ultrasensitive pH‐responsive degradability for the improvement of PDT as well as MRI.^[^ ^54^ ^]^ Benefiting from their inherently excellent properties such as adequate biodegradation, excellent biocompatibility, and pH‐controlled cargo release, calcium phosphate (CaP) nanoparticles have drawn booming research attention as one of the most advantageous nanomaterials.^[^ ^105^ ^]^ Consequently, Kataoka and co‐workers took advantage of pH‐sensitive CaP nanoparticles to confine Mn^2+^ ions, which would stay stable (less than 8% Mn^2+^ ions were released) at pH 7.4 but would disintegrate and release Mn^2+^ ions more rapidly (36% at pH 6.7, 71% at pH 6.5 and over 95% at pH 5) within 4 h (Figure 2C).^[^ ^106^ ^]^ Similarly, Huang et al. mineralized glucose oxidase with manganese–doped CaP to construct pH‐responsive theranostic nanoplatform for diagnosis and cascade reaction‐enhanced cooperative cancer therapy.^[^ ^107^, ^108^, ^109^, ^110^ ^]^ Other researchers also applied CaP to construct pH‐responsive theranostic nanoplatforms, such as loading photosensitizer and prodrug for amplified combination therapy,^[^ ^111^ ^]^ or encapsulating drug and contrast agents for MRI‐guided chemotherapy.^[^ ^112^ ^]^

Recently, manganese silicate NPs have attracted great interest because of their efficacy of T_1_ MRI contrast under slightly acidic and elevated glutathione conditions of the TME, which have been extensively reported as pH‐responsive T_1_ MRI contrast agents.^[^ ^113^, ^114^, ^115^, ^116^ ^]^ Moreover, manganese silicate‐based theranostic nanoplatforms were designed for MRI‐guided pH‐responsive delivery of drugs.^[^ ^117^, ^118^ ^]^

Arsenite (AsO_3_^3−^) can coprecipitate with Mn^2+^ cations at neutral conditions to form the manganese arsenite compound which can collapse in acidic environments, releasing Mn^2+^ ions and arsenite.^[^ ^119^ ^]^ Attracted by this fascinating property, some researchers encapsulated water‐insoluble manganese arsenite compounds into hollow silica NPs (denoted as MnAsO*_x_* @SiO_2_) to construct the arsenite loading and pH‐responsive release nanosystem monitored by activatable T_1_ MRI.^[^ ^120^, ^121^ ^]^

## 3 PH‐RESPONSIVE NANOPLATFORMS FOR MR IMAGING

Being an advantageous non‐invasive molecular imaging technology with non‐ionizing radiation, non‐limited penetration depth, and high spatial resolution, MRI possesses the great potential for disease diagnosis, such as peculiar tissue structure, impeded blood flow, and vascularization.^[^ ^122^, ^123^ ^]^ The MR signal intensity could be adjusted with contrast agents (CAs) by reducing the relaxation time of water protons to enhance the contrast effect. The main approach of tumor imaging is to employ NP‐based CAs to enhance the contrast between tumor sites and normal tissues, thus surmounting the inferior tumor recognition as well as the ordinary signal‐to‐noise ratio. Therefore, tumor‐targeting ligands which can specifically bind with tumor epitopes, could be installed onto the NPs to obtain an optimized tumor‐cell specificity.^[^ ^124^, ^125^ ^]^ In addition, to improve the specificity of MRI, nanoplatforms that could generate pH‐dependent MRI signals have also been developed. And there are mainly two approaches to construct such pH‐responsive nanoplatforms for MRI. One is to encapsulate MRI CAs within pH‐responsive components (e.g., block copolymers,^[^ ^23^, ^31^ ^]^ hydrogels,^[^ ^30^ ^]^ CaP^[^ ^106^, ^107^ ^]^) which would be affected by protons and specifically release the CAs into tumor sites. The other approach is to employ those MRI CAs that simultaneously possess pH‐responsiveness (e.g., Fe‐GA,^[^ ^71^ ^]^ LDHs,^[^ ^83^ ^]^ MnO_2_,^[^ ^91^, ^93^ ^]^ and Mn(II) salts^[^ ^103^, ^113^, ^121^ ^]^). Depending upon the magnetic susceptibilities, MRI contrast agents can be classified into two forms, that is, T_1_ positive and T_2_ negative.

### 3.1 T_1_ MRI contrast agents

For enhanced T_1_ MRI contrast, nanomaterials should possess multiple high‐spin metal cations exposed on the outside for promoted interaction with water protons in the surrounding environment.^[^ ^126^, ^127^ ^]^ That demands the application of smaller NPs with the higher surface‐to‐volume ratio. However, merely employing numerous small NPs is not consistent with the economical utilization of targeting components. As for large NPs, the metal cations hidden inside would not contribute to T_1_ MRI contrast.

As one of the most widely and clinically applied MRI CAs, paramagnetic gadolinium(III) chelates can improve the contrast effect via shortening the longitudinal relaxation time of the surrounding water protons beside Gd^3+^ ions.^[^ ^23^ ^]^ In spite of the broad prospect of MRI, the application of clinically approved Gd‐based CAs is hindered by the inherent low efficiency of CAs, which leads to the demand for high dose administration of CAs.^[^ ^64^ ^]^ Fortunately, the coupling of numerous Gd^3+^ ions to nanoscale platforms could lead to enhanced MR relaxivity. Consequently, nanocarriers that can considerably improve the Gd capacity and enhance the T_1_ relaxivity have been developed.^[^ ^28^, ^41^, ^64^ ^]^ Graphene oxide (GO), which has a highly distinct area as well as various functional groups, was employed to improve the capacity for Gd‐based CAs by coupling with gadolinium‐labeled dendrimers in the intraparticle areas to boost its T_1_ MRI contrast ability.^[^ ^28^ ^]^ In addition, Nguyen and co‐workers developed gadolinium‐conjugated polymer‐caged nanobins which possess a high localized concentration of Gd‐based CAs and greatly enhance the relaxivity of each NP compared with small‐molecule Gd CAs.^[^ ^64^ ^]^ In another case, hydroxylated mesoporous nanosilica covered with branched polyethylenimine conjugated with Gd‐DTPA was also reported, which possesses high payload of Gd and exhibits a high relaxivity per nanoparticle.^[^ ^41^ ^]^

The easy elimination by renal filtration and lack of specific tissue distribution for Gd‐based contrast agents could result in insufficient signal contrast between cancer site and its surrounding healthy tissues.^[^ ^112^ ^]^ To address these problems, the stimulus‐responsive nanoplatforms with selective signal enhancement in the tumor site need to be developed, which could potentially bring significant MR signal contrast between tumor tissue and normal tissue. Therefore, some researchers incorporated Gd‐based agents into pH‐sensitive nanoplatforms which possess pH‐dependent MR signal enhancement property. For instance, some pH‐sensitive materials (e.g. chitosan,^[^ ^50^ ^]^ block copolymer,^[^ ^23^, ^24^, ^25^, ^26^ ^]^ liposome,^[^ ^27^ ^]^ calcium phosphate^[^ ^112^ ^]^) have been applied to encapsulate Gd‐based CAs, which could efficiently release the Gd agents and significantly increase the accessibility of Gd agents to the surrounding water molecules under acidic conditions of the tumor. Consequently, the longitudinal relaxation time (T_1_) of surrounding water molecules would be drastically shortened, leading to a positive contrast enhancement in T_1_ MRI.^[^ ^128^ ^]^

**FIGURE 3:**
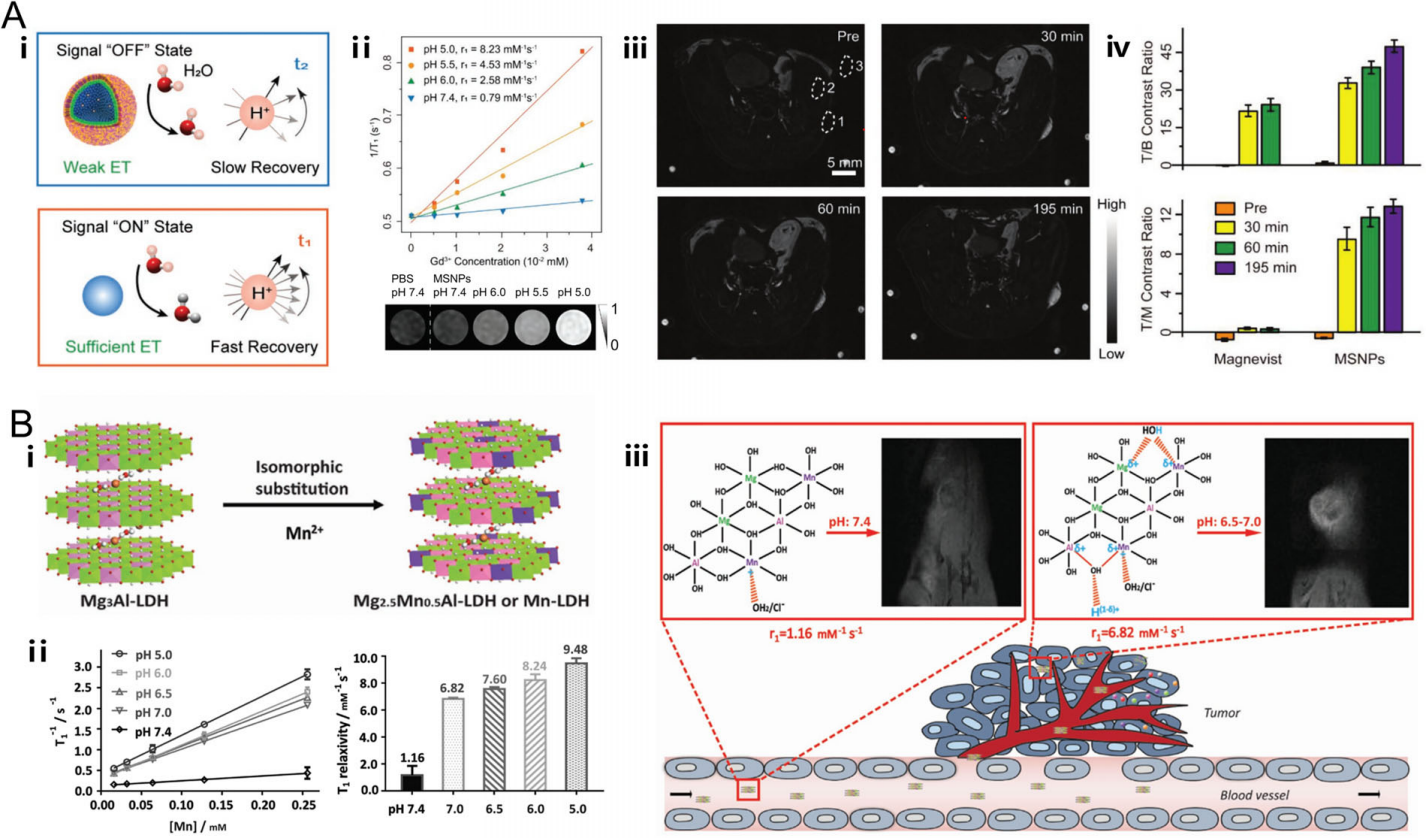
pH‐responsive nanoplatforms for T_1_ MR imaging. (A) Gd‐based T_1_ magnetic resonance imaging (MRI) CAs. The nanoplatform (MSNPs) is based on cell membrane‐coated NaGdF_4_‐CaCO_3_ nanocomposites. The spin interaction between crystal lattices and surrounding protons is structurally blocked by multilayers outside the T_1_ source (blue line). The energy transfer from protons to crystal lattices via spin‐lattice interaction is then activated due to the pH‐responsive capability of MSNPs under acidic microenvironments (orange line). The in vitro and in vivo MRI performance of MSNPs. Reproduced with permission.^[^ ^102^ ^]^ Copyright 2019, WILEY‐VCH Verlag GmbH & Co. KGaA. (B) Mn‐based T_1_ MRI CAs. The nanoplatform (Mn‐LDH) is based on Mn–doped layered double hydroxides. The in vitro and in vivo MRI performance of Mn‐LDH and schematic illustration of structure related multifunctional properties of Mn‐LDH. Reproduced with permission.^[^ ^83^ ^]^ Copyright 2017, WILEY‐VCH Verlag GmbH & Co. KGaA.

Apart from gadolinium chelates, some other Gd‐based inorganic contrast agents have been employed, such as lanthanide–doped NaGdF_4_ nanoparticles,^[^ ^12^, ^43^, ^67^, ^102^ ^]^ lanthanide–doped GdVO_4_,^[^ ^36^ ^]^ Gd–doped HfO_2_,^[^ ^129^ ^]^ Gd–doped hydroxyapatite nanorods,^[^ ^130^ ^]^ Gd_2_O_3_,^[^ ^97^, ^98^, ^131^ ^]^ which exhibited significant contrast enhancement performance in tumor visualization. Among these contrast agents, it is noteworthy that the MRI contrast ability of cell membrane‐coated NaGdF_4_‐CaCO_3_ nanocomposites (MSNPs) was originally compromised due to the spatial confinement of NaGdF_4_ nanoparticles and inadequate interactions between the crystal lattices and the protons. Nevertheless, under slightly acidic conditions of the tumor, the embedded CaCO_3_ NPs would produce CO_2_ bubbles and subsequently dissociate the nanoconjugate, leading to the recovered MRI signals and significant tumor/muscle contrast ratio compared with commercial contrast agent, Magnevist (Figure 3A).^[^ ^102^ ^]^

Mn^2+^ ions are representative T_1_ MRI CAs with labile water exchange, long relaxation time as well as high spin number.^[^ ^132^ ^]^ Mn‐incorporated nanoplatforms mostly possess pH‐responsive characteristic, which could efficiently release Mn^2+^ ions at a low pH for enhanced MR imaging. For instance, Wang and co‐workers encapsulated MnO nanoparticles into the silica nanoshell in which the MnO nanoparticles would be dissolved into Mn^2+^ when endocytosed into the acidic compartments of tumor cells.^[^ ^133^ ^]^ In view of the slight acidity‐induced dissolution of manganese oxide nanomaterials, it can be expected that the released Mn^2+^ would significantly enhance the T_1_ MRI contrast effect since the released Mn^2+^ ions possess larger accessibility to interact with H_2_O molecules in the surrounding environment.^[^ ^95^, ^134^ ^]^ Therefore, manganese oxide nanomaterials can serve as the pH‐sensitive MRI contrast agents for T_1_ MRI.^[^ ^95^, ^133^, ^134^, ^135^ ^]^ In addition, Xu et al. reported the Mn‐based LDH nanoparticles which have appealing T_1_ MR contrast ability under slightly acidic aqueous solution (*r*_1_ = 1.16 mM^−1^ s^−1^ at pH 7.4 while 6.82 mM^−1^ s^−1^ at pH 7.0) as well as the tumor site due to the protonation of hydroxyl groups around Mn^2+^ ions, which increased the coordinated number of water molecules with Mn^2+^ cations and enhanced the positive contrast in T_1_ MRI (Figure 3B).^[^ ^83^ ^]^ Some other Mn‐based nanoparticles (e.g., Mn‐based LDH,^[^ ^84^ ^]^ manganese oxide,^[^ ^95^, ^134^, ^135^ ^]^ manganese salt^[^ ^54^, ^103^, ^113^, ^120^ ^]^) also exhibited pH‐responsive property and could significantly enhance the MR signal contrast between tumor site and its surrounding healthy tissues.

Extremely small iron oxide nanoparticles (ESIONs) have drawn much attention for their capability as T_1_ MRI CAs since they possess suppressed magnetization and excellent T_1_ contrast ability. Meanwhile, ESIONs have good biocompatibility because the human body is capable of metabolizing iron ions, an innate element of hemoglobin.^[^ ^136^ ^]^ To specifically deliver ESIONs into tumor tissues, many researchers have designed and constructed pH‐sensitive nanocarriers (e.g., block copolymers,^[^ ^34^, ^137^ ^]^ mesoporous silica^[^ ^65^ ^]^) which could efficiently unload ESIONs to increase the accessibility to the surrounding water molecules under acidic conditions and specifically enhance the MRI contrast of tumor.

In addition, some iron coordinated complexes can also act as T_1_ MRI contrast agents^[^ ^69^, ^78^ ^]^ and some of them can even lead to an enhanced MR signal at a lower pH, which is mainly attributed to the increased accessibility of Fe^3+^ ions to the surrounding water molecules.^[^ ^71^, ^79^, ^80^ ^]^ For example, the coordinatively unsaturated Fe‐containing Fe^3+^/gallic acid complex can efficiently release Fe^3+^ and enhance the MR signal in the acidic environment of tumor in response to a lower pH.^[^ ^71^ ^]^ FeOOH clusters can respond to an acidic environment and release paramagnetic Fe^3+^ ions, leading to significant T_1_ MRI contrast enhancement.^[^ ^80^ ^]^ Besides, Fe^3+^/PDA complex also displayed pH‐activatable MRI contrast ability.^[^ ^79^ ^]^

### 3.2 T_2_ MRI contrast agents

**FIGURE 4:**
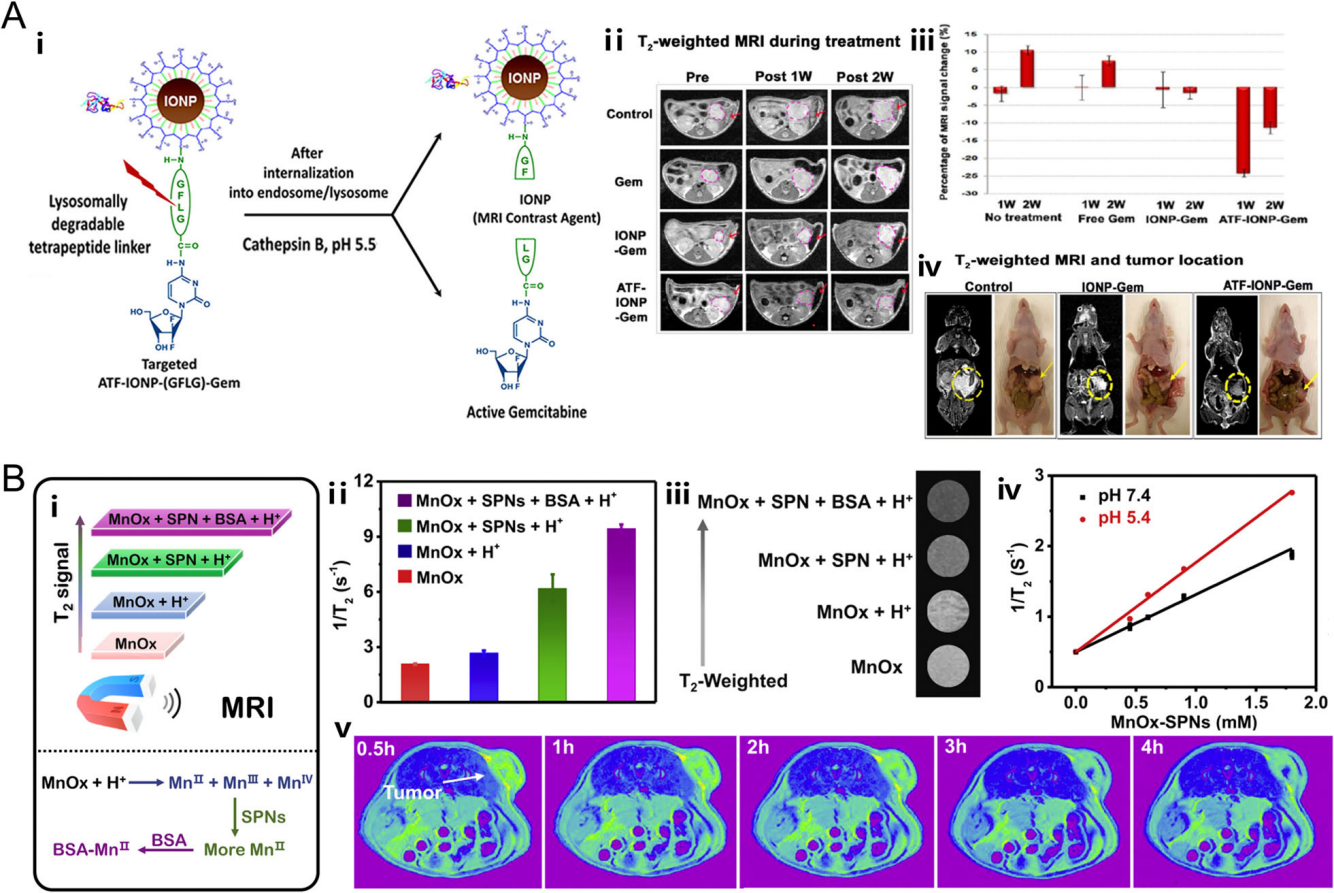
pH‐responsive nanoplatforms for T_2_ MR imaging. (A) FeOx‐based T_2_ magnetic resonance imaging (MRI) CAs. The nanoplatform (ATF‐IONP‐Gem) is based on amino‐terminal fragment (ATF) peptide‐conjugated iron oxide nanoparticles (IONPs). The in vivo MRI performance of nontargeted IONP‐Gem and ATF‐IONP‐Gem. Reproduced with permission.^[^ ^140^ ^]^ Copyright 2013, American Chemical Society. (B) MnO*_x_*‐based T_2_ MRI CAs. The nanoplatform (MnO*_x_*‐SPNs) is based on ultrathin MnO*_x_* nanosheets and semiconducting polymer nanoparticles (SPNs). Schematic illustration of MR signal amplification activated by introducing SPNs or BSA. The in vitro and in vivo MRI performance of MnO*_x_*‐SPNs. Reproduced with permission.^[^ ^151^ ^]^ Copyright 2020, Elsevier Inc.

Superparamagnetic iron oxide nanoparticles (SPIONs) have been widely employed for enhanced T_2_ MRI contrast due to their high magnetization values.^[^ ^138^ ^]^ Additionally, clusters of SPIONs present much better T_2_ contrast effect than separate NPs.^[^ ^139^ ^]^ Consequently, the gathering of iron oxide NPs with improved magnetization has attracted much attention owing to enhanced T_2_ MRI contrast as well as the economical utilization of targeting components. Following this strategy, Yang and co‐workers conjugated iron oxide nanoparticles (IONPs) with the amino‐terminal fragment (ATF) peptide, a natural target ligand, to provide MRI contrast enhancement of the tumor. The results of MRI signal change demonstrated that the existence of targeting ligands greatly enhanced the T_2_ MRI signal of the tumor site 1 week after administration (Figure 4A).^[^ ^140^ ^]^ In addition, some other nanoplatforms loaded with SPIONs and modified with a variety of targeting ligands (e.g., folic acid,^[^ ^46^, ^47^, ^51^, ^118^, ^141^, ^142^, ^143^, ^144^ ^]^ lactoferrin,^[^ ^145^ ^]^ peptide,^[^ ^40^, ^57^, ^58^, ^59^, ^62^, ^146^, ^147^ ^]^ antibody^[^ ^148^, ^149^ ^]^) have also been developed to facilitate the accumulation into tumor tissues and provide the contrast enhancement in T_2_ MRI.

Among the researches of MRI CAs, many efforts have been concentrated on the improvement of MOFs‐based CAs. Those nanomaterials could easily transport paramagnetic Fe^3+^ ions in large amounts which would generate enhanced contrast of the pathological tissue.^[^ ^74^ ^]^ It is notable that MOFs of Fe^3+^ (e.g., MIL‐100) have been widely reported to exhibit excellent payload for anticancer drugs and T_2_ MRI contrast ability, which attracted much interest in biomedical application field.^[^ ^52^, ^75^, ^150^ ^]^

Apart from iron‐based T_2_ MRI contrast agents, our group recently reported on a manganese‐based nanosystem (denoted as MnO*_x_*‐SPNs) consisting of ultrathin MnO*_x_* nanosheets and semiconducting polymer nanoparticles (SPNs), which could potentially act as pH‐activatable T_2_ MRI contrast agent (Figure 4B).^[^ ^151^ ^]^ More specifically, MnO*_x_* nanosheets would dissolve into Mn^2+^, Mn^3+,^ and Mn^4+^ under acidic conditions, in which the Mn ions with higher valance states would be probably reduced into Mn^2+^ by SPNs. Moreover, when Mn^2+^ combined with bovine serum albumin (BSA), the Mn‐BSA exhibited an even better T_2_ contrast effect, thus achieving the multi‐step signal amplification.

### 3.3 T_1_‐T_2_ dual modal MRI contrast agents

Despite every MRI mode having its own advantage, sometimes a single MRI mode cannot meet the high diagnostic requirements because each form of CAs has its inherent shortcomings and the medical diagnostic results could be deceptive merely by employing the single imaging modality.^[^ ^152^ ^]^ For instance, the T_2_ negative signals obtained with CAs are frequently confused with the signals from metal deposits, bleeding, or calcification.^[^ ^153^ ^]^ This restriction can be mended by integrating T_1_ and T_2_ MR imaging modalities into a single nanoplatform since it could produce diagnostic results with great accuracy benefiting from high tissue resolution provided by T_1_ MRI as well as high practicability on detecting lesions provided by T_2_ MRI.^[^ ^154^ ^]^ Consequently, MRI contrast agents with T_1_‐T_2_ dual modal functions have attracted considerable interest.

**FIGURE 5:**
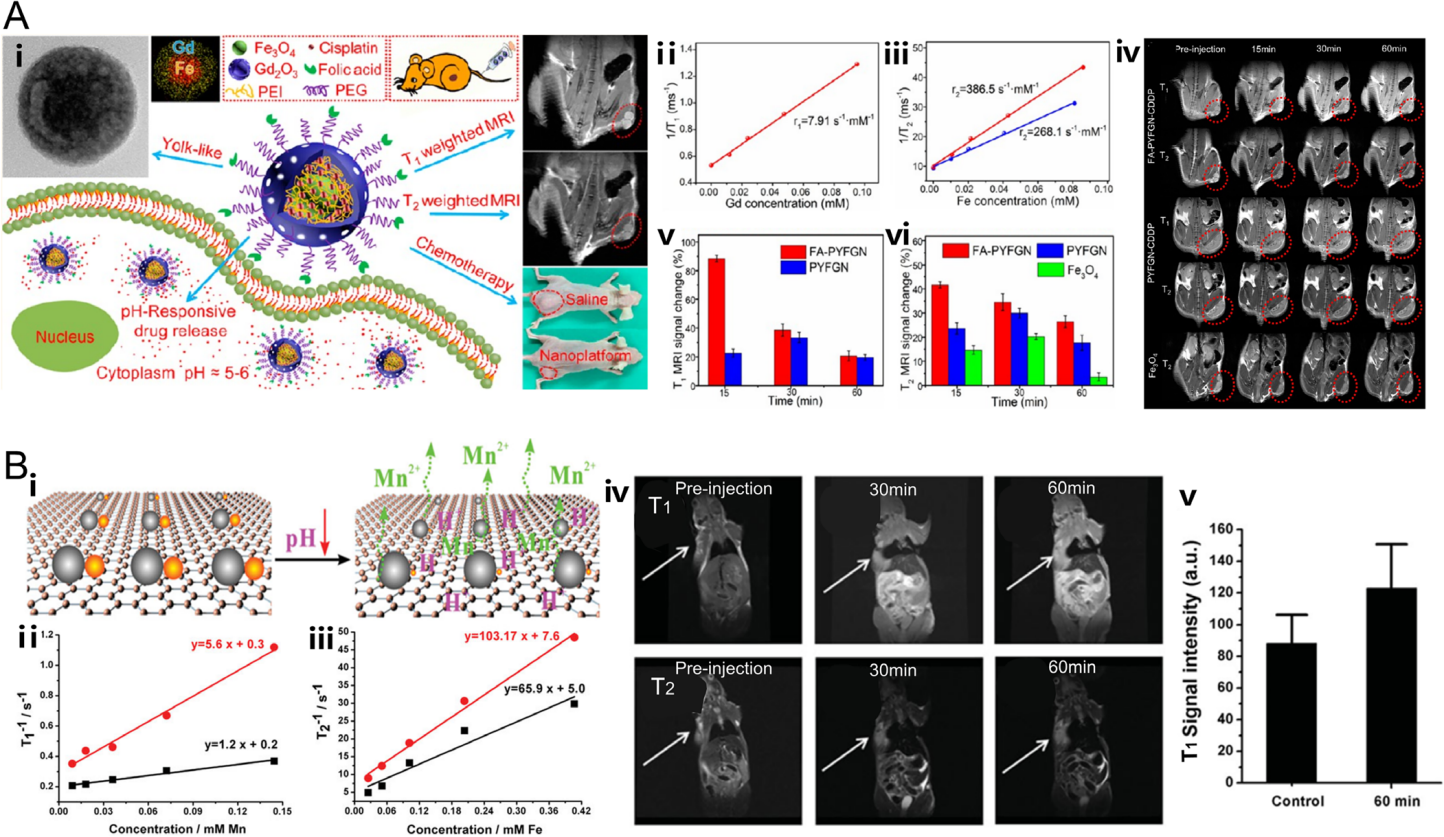
pH‐responsive nanoplatforms for T_1_‐T_2_ dual modal MR imaging. (A) Gd/Fe‐based magnetic resonance imaging (MRI) CAs. The nanoplatform (FA‐PYFGN‐CDDP) is based on folic acid (FA) functionalized SPIONs‐Gd_2_O_3_ core–shell nanocomposites. The in vitro MRI performance of FA‐PYFGN‐CDDP and Fe_3_O_4_. And the in vivo MRI performance of FA‐PYFGN‐CDDP, nontargeted PYFGN‐CDDP, and Fe_3_O_4_. Reproduced with permission.^[^ ^156^ ^]^ Copyright 2017, American Chemical Society. (B) Mn/Fe‐based MRI CAs. The nanoplatform (Fe_3_O_4_/MnO*_x_*‐GO) is based on Fe_3_O_4_ and MnO*_x_* co‐integrated graphene oxide (GO) nanosheets. The in vitro (black line: pH 7.4 and red line: pH 6.0) and in vivo MRI performance of Fe_3_O_4_/MnO*_x_*‐GO. Reproduced with permission.^[^ ^29^ ^]^ Copyright 2014, WILEY‐VCH Verlag GmbH & Co. KGaA.

Recently, many T_1_‐T_2_ dual modal CAs have been developed, such as gadolinium–doped IONPs,^[^ ^155^ ^]^ which exhibited excellent T_1_‐T_2_ dual modal MR contrast enhancement both in vitro and in vivo. Wu et al. developed the polyethylene glycol and folic acid (FA) functionalized nanocomposites (FA‐PYFGN‐CDDP) with the core of SPIONs and the shell of mesoporous Gd_2_O_3_ layer, which have the *r*_1_ value of 7.91 mM^−1^ s^−1^ and *r*_2_ value of 386.5 mM^−1^ s^−1^. Besides, the MR images of mice showed that the maximum signal change for both T_1_ and T_2_ MRI was obtained 15 min after administration of FA‐PYFGN‐CDDP (Figure 5A).^[^ ^156^ ^]^ In addition, some other researchers have successfully integrated SPIONs and Mn‐based agents into a single nanoplatform acting as T_2_ and T_1_ contrast agents respectively.^[^ ^115^, ^157^ ^]^ Intriguingly, both dual modal contrast agents presented enhanced T_1_ and T_2_ MR signals under acidic conditions of tumor due to the pH‐dependent release of T_1_ MRI contrast agents. For instance, Shi et al. realized the co‐integration of SPIONs and MnO*_x_* NPs onto exfoliated graphene oxide nanosheets (denoted as Fe_3_O_4_/MnO*_x_*‐GO) through a facile two‐step double redox strategy, in which the MnO*_x_* nanoparticles could be dissolved into Mn^2+^ under acidic conditions. Notably, both T_1_ and T_2_ MR signal enhancements were observed both in vitro and in vivo in an acidic environment (Figure 5B).^[^ ^29^ ^]^ In another case, an intelligent nanoflower composite with superparamagnetic Fe_3_O_4_ nanoclusters as the core and MnO_2_ nanosheets as the outer shell was also developed for T_1_‐T_2_ dual modal imaging.^[^ ^158^ ^]^

Apart from integrating two different kinds of contrast agents into one nanoplatform, some other researchers have applied one single kind of nanomaterial (e.g., R‐PtWMn,^[^ ^159^ ^]^ Prussian blue,^[^ ^76^, ^88^ ^]^ NaMnF_3_,^[^ ^39^ ^]^ Mn_3_[Co(CN)_6_]_2_^[^ ^77^ ^]^) which can act as both T_1_ and T_2_ MRI CAs to endow the system with dual modal imaging ability.

## 4 PH‐RESPONSIVE THERANOSTIC NANOPLATFORMS FOR MRI‐GUIDED SINGLE MODE THERAPY

Nowadays, cancer is one of the most severe illnesses menacing physical health of citizens.^[^ ^42^ ^]^ To fight against cancer, a wide range of treatment modes have emerged, such as chemotherapy, radiotherapy, PDT, chemodynamic therapy, hyperthermia, immunotherapy, etc. Notably, all the therapeutic modes discussed below were carried out with pH‐responsive nanoplatforms and under MRI guidance, which to some degree improved the specificity and accuracy of the treatment.

### 4.1 Chemotherapy

**FIGURE 6:**
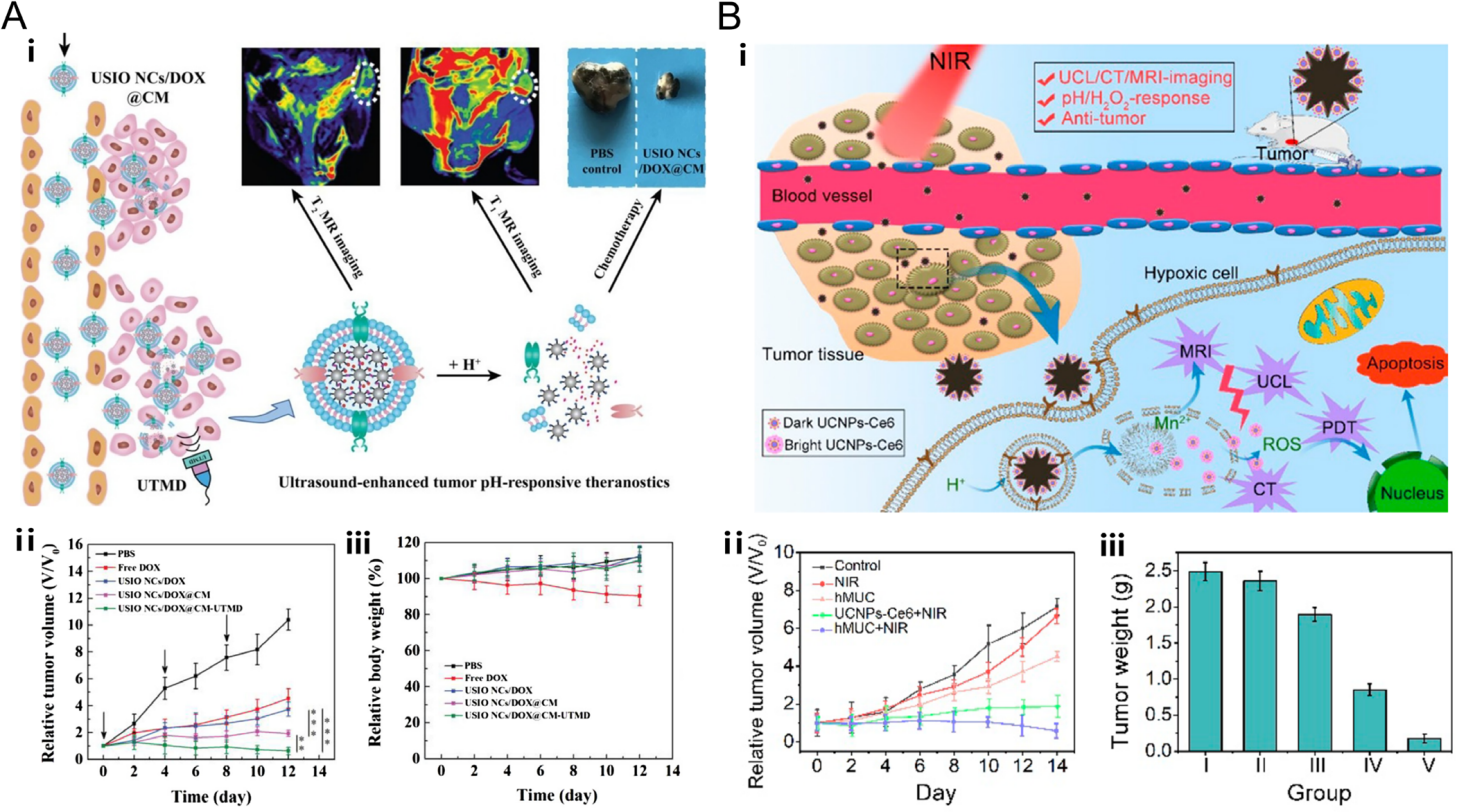
pH‐responsive nanoplatforms for (A) chemotherapy. The nanoplatform (USIO NCs/DOX@CM) is based on cell membrane (CM)‐coated and DOX‐loaded ultrasmall iron oxide nanoclusters (USIO NCs). Schematic illustration for UTMD‐promoted delivery of USIO NCs/DOX@CM to achieve pH‐responsive tumor theranostics. The therapeutic outcomes of USIO NCs/DOX@CM‐UTMD and other groups. Reproduced with permission.^[^ ^161^ ^]^ Copyright 2020, Elsevier Ltd. (B) Photodynamic therapy. The nanoplatform (hMUC) is based on honeycomb MnO*_x_* NPs and Ce6‐sensitized up‐conversion nanoparticles (UCNPs). Schematic illustration of the systemic delivery of hMUC in vivo, and corresponding diagnosis and therapy for tumor. The therapeutic outcomes of hMUC+NIR and other groups. Reproduced with permission.^[^ ^96^ ^]^ Copyright 2018, American Chemical Society.

Among the various treatment approaches, chemotherapy is essential because it can be easily employed in diverse types of cancers. Fortunately, the current chemotherapeutic drugs are generally effective to kill various types of tumor cells.^[^ ^160^ ^]^ However, the primary issue in the clinical chemotherapy is the fatal side effects of the anticancer drugs which are induced by the non‐specific attack of the drug molecules toward normal cells.^[^ ^160^ ^]^ A reasonable method to overcome these shortcomings is targeted delivery of chemotherapeutic drugs specifically into tumor cells. By taking advantage of the slightly acidic characteristic in tumor tissues, nanotechnology‐mediated pH‐responsive drug delivery systems have emerged. For example, Shen and co‐workers developed the pH‐responsive nanoclusters (USIO NCs/DOX@CM) which could be dissociated into ultrasmall IONPs with dynamic T_2_/T_1_ MRI and unload the anticancer drug DOX efficiently. Moreover, through the ultrasound‐targeted microbubble destruction (UTMD) technology, the tumor accumulation and penetration of USIO NCs/DOX@CM would be enhanced, leading to significant tumor suppressive effect. Notably, pH‐triggered disassembly of nanoplatforms to release drug and brighten T_1_ MRI, thus MRI signals were used to monitor the accumulation and release of drug. Compared to the free DOX treatment, the USIO NCs/DOX@CM‐UTMD treatment showed great biosafety with no body weight loss recorded (Figure 6A).^[^ ^161^ ^]^ Additionally, other pH‐responsive drug delivery systems such as mesoporous silica,^[^ ^41^, ^65^, ^67^, ^80^, ^162^ ^]^ MOFs,^[^ ^74^, ^87^, ^163^ ^]^ polymeric micelles,^[^ ^34^, ^78^, ^164^, ^165^ ^]^ nanohydrogel,^[^ ^36^, ^166^ ^]^ MnO_2_ nanosheets,^[^ ^91^, ^167^ ^]^ graphene oxide^[^ ^28^ ^]^ and liposomes^[^ ^27^, ^62^ ^]^ have also been developed, showing huge application potential in cancer treatment. Moreover, the therapeutic process could be monitored by MRI, which further improves the specificity of cancer treatment.

Benefiting from these targeted drug delivery systems, a variety of chemotherapeutic drugs were specifically unloaded into the slightly acidic tumor sites. Among these chemotherapeutic drugs, doxorubicin is most widely employed and it can intercalate between base pairs of the DNA helix, hence impeding DNA replication and eventually hindering protein synthesis.^[^ ^12^, ^91^, ^168^ ^]^ Besides, many other kinds of chemotherapeutic drugs (e.g., noscapine,^[^ ^169^ ^]^ artesunate,^[^ ^77^ ^]^ sorafenib,^[^ ^60^, ^148^, ^164^ ^]^ cisplatin,^[^ ^156^, ^170^ ^]^ gemcitabine,^[^ ^64^ ^]^ artemisinin,^[^ ^118^ ^]^ paclitaxel,^[^ ^62^, ^171^ ^]^ 5‐fluorouracil,^[^ ^163^, ^172^ ^]^ gemcitabine^[^ ^140^ ^]^) have also been applied for chemotherapy.

It's well‐confirmed that each type of anticancer drug has its distinctive growth inhibition mechanism through the period of cell cycle. However, the application of one type of chemotherapeutic drugs is insufficient for the treatment of tumors because their complicated structure and heterogeneity could lead to significant drug resistance. Therefore, the co‐delivery of multi chemotherapeutic drugs through nanoplatforms has been developed and exhibited greater toxicity toward various tumor cells on account of their different anticancer activity through different period of tumor development than the single drug.^[^ ^173^ ^]^ Qiao et al. encapsulated DOX, camptothecin, and combretastatin A4 in the same nanoparticles which could be effectively released to the corresponding active sites in time and space for tumor combined chemotherapy.^[^ ^42^ ^]^ Additionally, the combinations of DOX and rhodamine B,^[^ ^174^ ^]^ DOX, and colchicine^[^ ^28^ ^]^ were also employed for synergistic chemotherapy.

### 4.2 PDT

As a clinically approved non‐invasive therapeutic method, PDT utilizes a photosensitizer, oxygen molecules, and an appropriate exciting light through the production of cytotoxic reactive oxygen species (ROS) to destroy biomolecules such as DNAs and biological membranes inside tumor cells.^[^ ^54^ ^]^ The primary obstacle to PDT is hypoxia in the TME.^[^ ^96^ ^]^ It is well‐established that the rapid‐growing tumor cells as well as the abnormal and generally twisted blood vessels inside solid tumors are accountable for inadequate O_2_ supply, higher H_2_O_2_ level, and acidity in the solid tumors.^[^ ^175^ ^]^ To ameliorate the oxygen‐deprived condition and further improve the curative effect of PDT, many efforts have been made to construct nanoplatforms that could realize self‐sufficiency of O_2_, delivery, or generation of abundant O_2_ inside the tumor to overcome hypoxia. Going down this trend, several researchers discovered that manganese dioxide nanomaterials possess TME‐responsive and TME‐modulating properties, which could catalyze the decomposition of H_2_O_2_ into oxygen and water in the TME.^[^ ^92^ ^]^ Consequently, MnO_2_ nanomaterials can act as the O_2_‐producing and H_2_O_2_‐depleting agent for the improvement of the PDT performance. Based on this finding, Yang and co‐workers developed an intelligent pH‐controllable and H_2_O_2_‐responsive nanosystem (hMUC) based on honeycomb MnO*_x_* NPs and chlorin e6 (Ce6)‐sensitized up‐conversion nanoparticles (UCNPs). When irradiated by tissue‐penetrable 808 nm laser, UCNPs would emit visible photons with higher‐energy which could be absorbed by Ce6 to produce cytotoxic ROS, thereby inducing PDT process naturally and suppressing tumor growth prominently (Figure 6B).^[^ ^96^ ^]^ Similarly, Ge et al. fabricated carbon dots/manganese dioxide nanocomposites, which exhibited enhanced ROS generation and significantly promoted PDT efficacy.^[^ ^176^ ^]^ In the meantime, the treatment of cancer would be more precise and specific under the guidance of MRI.^[^ ^96^, ^176^ ^]^

In addition to the strategy of improving tumor hypoxia, the PDT efficacy could also be enhanced through blocking the hypoxia‐inducible factor‐1α/vascular endothelial growth factor (HIF‐1α/VEGF) pathway.^[^ ^54^ ^]^ Based on this strategy, Zhang and co‐workers applied photosensitizer and acriflavine(ACF)‐loaded porous manganese phosphate (PMP) NPs as the photoactivable synergistic system which imparts ROS triggered cytotoxicity in coordination with HIF‐1α /VEGF inhibitor which prevents tumor growth and development as well as treatment escape signaling pathway. Upon the stimulus of the tumor acidic microenvironment, the PMP NPs would disintegrate to release Mn^2+^ for MRI and the photosensitizer Ce6 would be released to produce ROS under irradiation while ACF blocks the HIF‐1α /VEGF pathway during the burst release of VEGF in tumor triggered by PDT, leading to improved curative effect.^[^ ^54^ ^]^

### 4.3 Photothermal therapy

**FIGURE 7:**
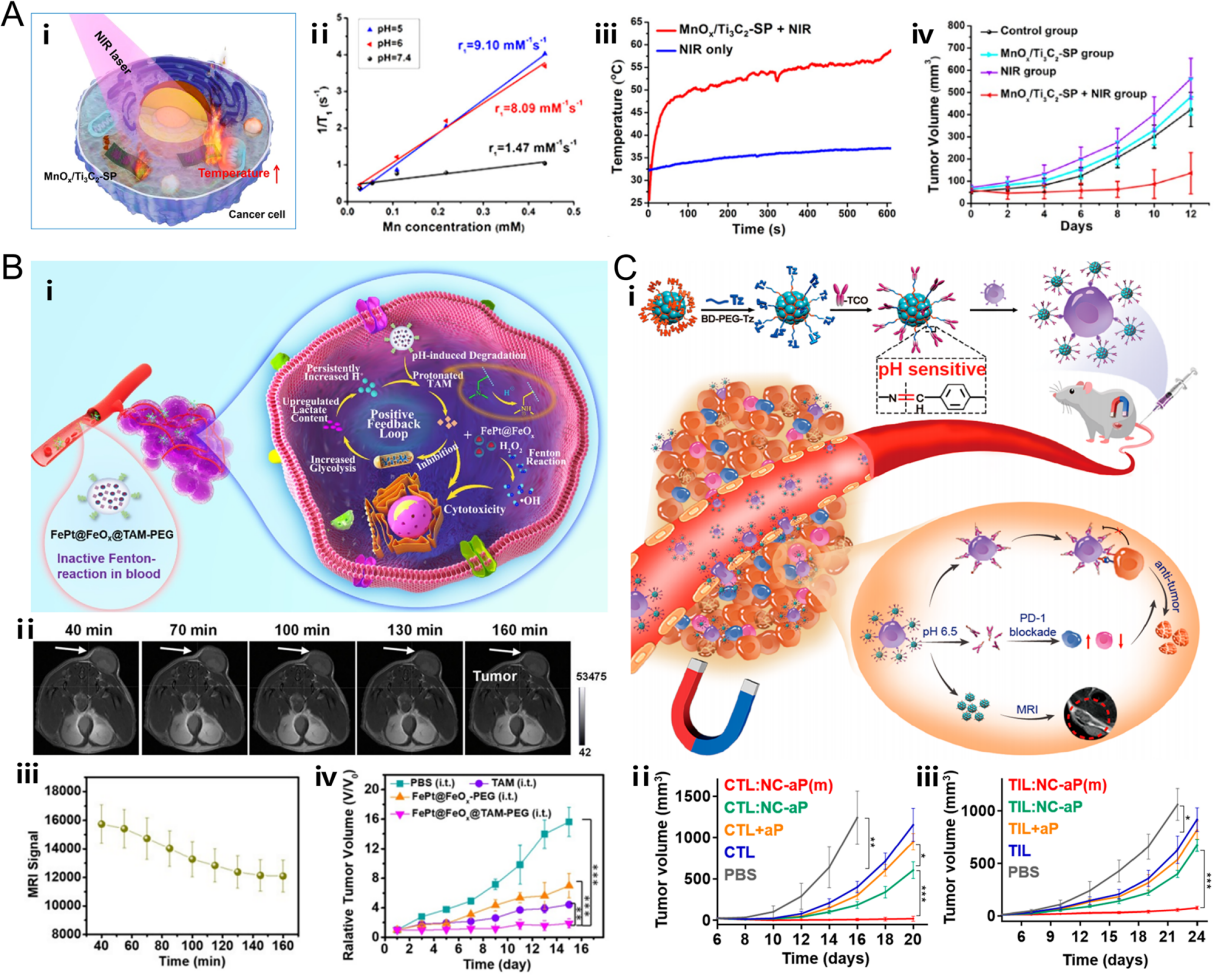
pH‐responsive nanoplatforms for (A) photothermal therapy. The nanoplatform (MnO*_x_*/Ti_3_C_2_−SP) is based on soybean phospholipid (SP)‐modified MnO*_x_*/Ti_3_C_2_ nanosheets. Schematic illustration of MnO*_x_*/Ti_3_C_2_−SP nanosheets as the photothermal agents for cancer cell ablation. The magnetic resonance imaging (MRI) and PTT performance of MnO*_x_*/Ti_3_C_2_−SP. Reproduced with permission.^[^ ^135^ ^]^ Copyright 2017, American Chemical Society. (B) Chemodynamic therapy. The nanoplatform (FePt@FeO*_x_* @TAM‐PEG) is based on core–shell structured FePt@FeO*_x_* and pH‐sensitive drug tamoxifen (TAM). Schematic illustration of acidity‐unlocked FePt@FeO*_x_* @TAM‐PEG with positive feedback loop promoting Fenton‐like reactions for self‐boosting tumor specific chemodynamic therapy. The MRI and CDT performance of FePt@FeO*_x_* @TAM‐PEG. Reproduced with permission.^[^ ^181^ ^]^ Copyright 2021, Wiley‐VCH GmbH. (C) Immunotherapy. The nanoplatform (NC‐aP) is based on PD‐1 antibody‐conjugated Fe_3_O_4_ nanoclusters. Schematic illustration of magnetic nanoclusters armed with PD‐1 antibody improved adoptive T cell therapy for solid tumors. The therapeutic outcomes of NC‐aP under magnetic field and other groups. Reproduced with permission.^[^ ^184^ ^]^ Copyright 2019, American Chemical Society.

As a promising therapeutic strategy for cancer, photothermal therapy (PTT) has attracted much interest on account of its minimal damage to healthy tissues and great ablation efficiency, which is generally induced by near‐infrared (NIR) laser to produce heat for the thermal ablation of the tumor in situ.^[^ ^177^, ^178^, ^179^ ^]^ For the purpose of optimizing the PTT efficacy, diverse photothermal‐transducing agents have been employed including 2D titanium carbide (Ti_3_C_2_),^[^ ^135^ ^]^ PDA,^[^ ^79^, ^104^ ^]^ and polypyrrole.^[^ ^31^ ^]^ Particularly, MRI‐guided PTT has exhibited great potential as a promising theranostic strategy for clinical application. MRI can recognize the location and size of the tumor while the NIR laser can be specifically applied onto the tumor site. For instance, Chen et al. designed the MnO*_x_*/Ti_3_C_2_ nanocomposite (MnO*_x_*/Ti_3_C_2_‐SP) for pH‐activated MRI and PTT. Under the NIR light irradiation for 10 min, the tumor temperature increased significantly from 25°C to 60°C with the injection of MnO*_x_*/Ti_3_C_2_‐SP, which is high enough to destroy tumor cells and suppress tumor growth (Figure 7A).^[^ ^135^ ^]^ Moreover, by taking advantage of pH‐dependent MR contrast enhancement, a more precise and satisfactory theranostic process would be obtained. Consequently, pH‐activatable MRI‐guided PTT could realize the optimized specificity and minimal damage to normal tissues.^[^ ^31^, ^79^, ^104^, ^135^ ^]^

### 4.4 Chemodynamic therapy

Chemodynamic therapy (CDT), as an emerging alternative therapeutic modality, employs CDT agents to transform H_2_O_2_ into the hydroxyl radical (·OH), the most damaging ROS through Fenton/Fenton‐like reactions, thereby triggering cell apoptosis and necrosis.^[^ ^99^, ^180^ ^]^ Based on this treatment strategy, our group recently encapsulated core–shell structured FePt@FeO*_x_* NP and pH‐sensitive drug tamoxifen (TAM) simultaneously into polymeric matrix (denoted as FePt@FeO*_x_* @TAM‐PEG) for tumor‐specific MRI‐guided CDT. Notably, the catalytic activity of FePt@FeO*_x_* is initially blocked in normal tissues. However, the TAM could be protonated and released under acidic conditions of tumor, which is capable of inhibiting mitochondrial complex I and thereby upregulating the lactate content and intracellular H^+^. Consequently, FePt@FeO*_x_* nanocatalyzers would be more efficiently released and exert the improved Fenton‐like reaction under more acidic conditions, which significantly suppressed the growth of tumor (Figure 7B).^[^ ^181^ ^]^ Additionally, Hou et al. fabricated the core–shell NPs with a core of magnetic Fe_5_C_2_ and a shell of pH‐responsive iron oxide coating, which could disintegrate and release ferrous ions under acidic conditions to convert H_2_O_2_ into ·OH radicals, thus efficiently inhibiting the proliferation of tumor cells. Furthermore, the dissolution of iron oxide coating simultaneously decreased the T_2_ MR signal and increased the T_1_ MR signal, and this T_2_/T_1_ switching feature offers opportunity for visualizing the release of Fe^2+^ and the production of ROS for the surveillance of tumor treatment.^[^ ^99^ ^]^

### 4.5 Immunotherapy

Cancer immunotherapy has markedly shifted the paradigm of therapeutic strategy for cancer in the past two decades.^[^ ^182^ ^]^ Among the immunotherapeutic methods, an advantageous approach is to directly inject immunoadjuvants into the tumor sites to in situ induce cancer vaccination following immunogenic cell death treatments.^[^ ^183^ ^]^ The cancer vaccination attempts to convey the local tumor antigens to antigen presenting cells, especially to the dendritic cells, by a more effective pattern for the purpose of priming the antigen‐specific CD8+ T cells and realizing the production of CD8+ T cells in abundance to destroy tumor cells.^[^ ^183^ ^]^ Based on this strategy, Wang et al. employed the pH‐responsive hollow Gd_2_O_3_ nanospheres, which tend to conjugate with various biomolecules due to the existence of surface charge, to efficiently deliver F‐OVA, a model tumor antigen into tumor tissues for immunotherapy under the surveillance of pH‐dependent MRI.^[^ ^98^ ^]^ In addition, to promote the performance of adoptive T‐cell therapy, an immune‐based approach, Xie et al. developed magnetic nanoclusters conjugated with PD‐1 antibodies through pH‐responsive benzoic‐imine bond (denoted as NC‐aP), which could bind with effector T cells (e.g., CTLs, TILs) owing to their PD‐1 expression. Subsequently, both effector T cells and NC‐aP could be recruited into tumor sites under external magnetic field with MRI guidance. In an acidic environment of the tumor, the PD‐1 antibody would be released for PD‐1 blocking, which could work in a synergistic manner with the adoptive T cells. As a result, when exposed to an external magnetic field, the development of E.G7 tumor model would be completely suppressed in the CTL:NC‐aP(m) group, and the development of 4T1 tumor model would be almost totally inhibited in the TIL:NC‐aP(m) group (Figure 7C).^[^ ^184^ ^]^

## 5 PH‐RESPONSIVE THERANOSTIC NANOPLATFORMS FOR MRI‐GUIDED COMBINED THERAPY

Owing to the unsatisfactory curative effects obtained from single mode therapy, a synergistic treatment modality combining different drugs and mechanisms with improved performance for cancer therapy is exceedingly attractive. Furthermore, with the incorporation of MRI contrast agents, these pH‐responsive nanoplatforms exhibit even more precise and satisfactory therapeutic effects under the guidance of MRI.

### 5.1 Chemotherapy/PTT

The combination of PTT and chemotherapy has been widely established to be capable of achieving satisfactory therapeutic effects,^[^ ^69^, ^75^, ^76^, ^185^ ^]^ since chemotherapeutic drugs can be efficiently unloaded under high temperature and constantly generate the chemotherapeutic effect for the long term.^[^ ^147^, ^186^, ^187^, ^188^ ^]^ Additionally, when combined with chemotherapy, PTT could cooperatively improve the treatment effect, e.g., promotion of cell membrane permeability, enhanced accumulation of the NPs in tumor sites, and improvement of drug toxic effect.^[^ ^75^ ^]^ Furthermore, when combined with MRI‐based diagnostics, the precision, and specificity of the synergistic treatment would be significantly improved. In order to take advantage of these merits, Zhu and co‐workers constructed the redox‐sensitive disulfide linkers equipped Fe_3_O_4_@PDA NPs coated by hyaluronic acid, which were subsequently deposited with the chemotherapeutic drug, DOX. Under the irradiation of NIR light, PTT would be activated to produce heat for the thermal ablation of tumors. Meanwhile, the drug release would be induced by acidity, glutathione, and high temperature, thus realizing the synergistic application of PTT and chemotherapy.^[^ ^189^ ^]^

**FIGURE 8:**
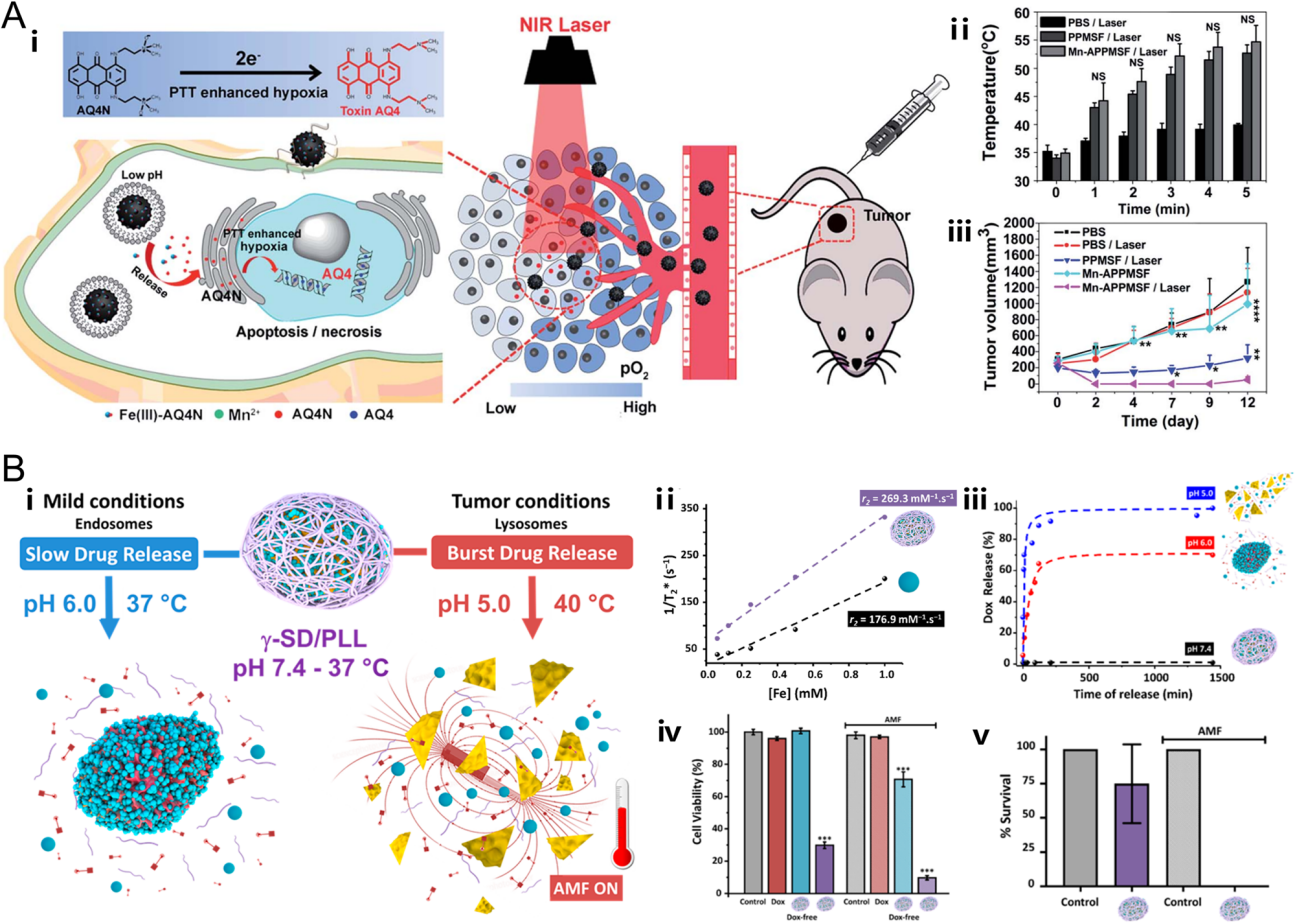
pH‐responsive nanoplatforms for combined therapy. (A) Chemotherapy and photothermal therapy. The nanoplatform (Mn‐APPMSF) is based on Fe(III)‐AQ4N prodrugs and Mn(II)‐loaded semiconducting polymer dots‐hybridized mesoporous silica. Schematic illustration of the functional principle of Mn‐APPMSF in tumor microenvironments. The PTT performance and therapeutic outcomes of Mn‐APPMSF/laser and other groups. Reproduced with permission.^[^ ^190^ ^]^ Copyright 2018, The Royal Society of Chemistry. (B) Chemotherapy and magnetic hyperthermia. The nanoplatform (γ‐SD/PLL) is based on covalent organic frameworks‐coated γ‐Fe_2_O_3_ NPs. Schematic representation of magnetically induced heat‐ and acid‐triggered Dox release from γ‐SD/PLL in conditions that mimic cancer cell physiology. The magnetic resonance imaging performance, DOX release profiles, and therapeutic outcomes of γ‐SD/PLL. Reproduced with permission.^[^ ^192^ ^]^ Copyright 2020, American Chemical Society.

Additionally, some researchers discovered that hyperthermia (*T* > 46°C) triggered by PTT would aggravate the hypoxia level in TME, leading to overexpressed hypoxia inducible factor‐1 (HIF‐1).^[^ ^190^ ^]^ It is noteworthy that banoxantrone (AQ4N) is a novel bio‐reductive prodrug showing excellent anticancer activity under hypoxic conditions through an enzymatic process via bio‐reduction to its metabolite AQ4.^[^ ^191^ ^]^ Based on this finding, Liu and co‐workers integrated the pH/hypoxia‐inducible Fe(III)‐AQ4N prodrugs and semiconducting polymer dots (denoted as Mn‐APPMSF), in which the Fe(III)‐AQ4N prodrug would be cleaved under acidic conditions of tumor, for programmable triggered and MRI‐guided photothermal‐chemotherapy. Upon NIR laser irradiation, the tumor temperature of Mn‐APPMSF injected mice could rise about 20°C in 5 min, which would lead to efficient cancer cell destruction via local hyperthermia and aggravate the tumor hypoxia level. Interestingly, the enhanced hypoxia could further accelerate the reduction of AQ4N to greatly improve the curative effects and significantly inhibit tumor growth (Figure 8A).^[^ ^190^ ^]^

### 5.2 Chemotherapy/magnetic hyperthermia

The combination of chemotherapy and magnetic hyperthermia (MH) is under research for the effective alleviation of tumors.^[^ ^15^, ^29^, ^173^, ^192^ ^]^ MH possesses great capability for heat‐activated ablation of tumor tissues at 43–45°C under external AC magnetic field. Moreover, it's able to enhance the drug toxicity in drug‐resistant cancer cells.^[^ ^173^ ^]^ Fe_3_O_4_ nanoparticles have been widely employed as the nanoconverter for MH to produce localized heat from the input of external magnetic energy as a result of their magnetically‐induced hyperthermia capability.^[^ ^15^, ^173^ ^]^ To further improve the hyperthermia capability of magnetic nanomaterials, the magnetic iron oxide (γ‐Fe_2_O_3_) NPs coated covalent organic frameworks (COFs) were also applied as MH agents to effectively generate more heat (48°C) when exposed to the AC magnetic field (AMF) because the porous structure of COFs could facilitate the heat conduction. Moreover, the nanoplatform (denoted as γ‐SD/PLL) exhibited efficient capacity for anticancer drug Doxorubicin and would disintegrate in the acidic environment for rapid drug release and MRI signal production, which achieved combined chemo‐thermotherapy under the surveillance of MRI (Figure 8B).^[^ ^192^ ^]^ Additionally, Shi et al. integrated magnetic Fe_3_O_4_ NPs onto exfoliated GO nanosheets, which possess the high heat transfer ability/thermal conductivity for synergistic improvement of MH, to construct a nanosystem that can be applied for MH upon exposure to external magnetic field. Meanwhile, GO nanosheets can efficiently load aromatic anticancer drugs (e.g., DOX) through supramolecular π stacking as well as MnO*_x_* NPs, which both exhibited pH‐responsive property, thus making this nanosystem capable for pH‐activatable MRI‐guided synergistic chemo‐thermotherapy.^[^ ^29^ ^]^

### 5.3 Chemotherapy/PDT

**FIGURE 9:**
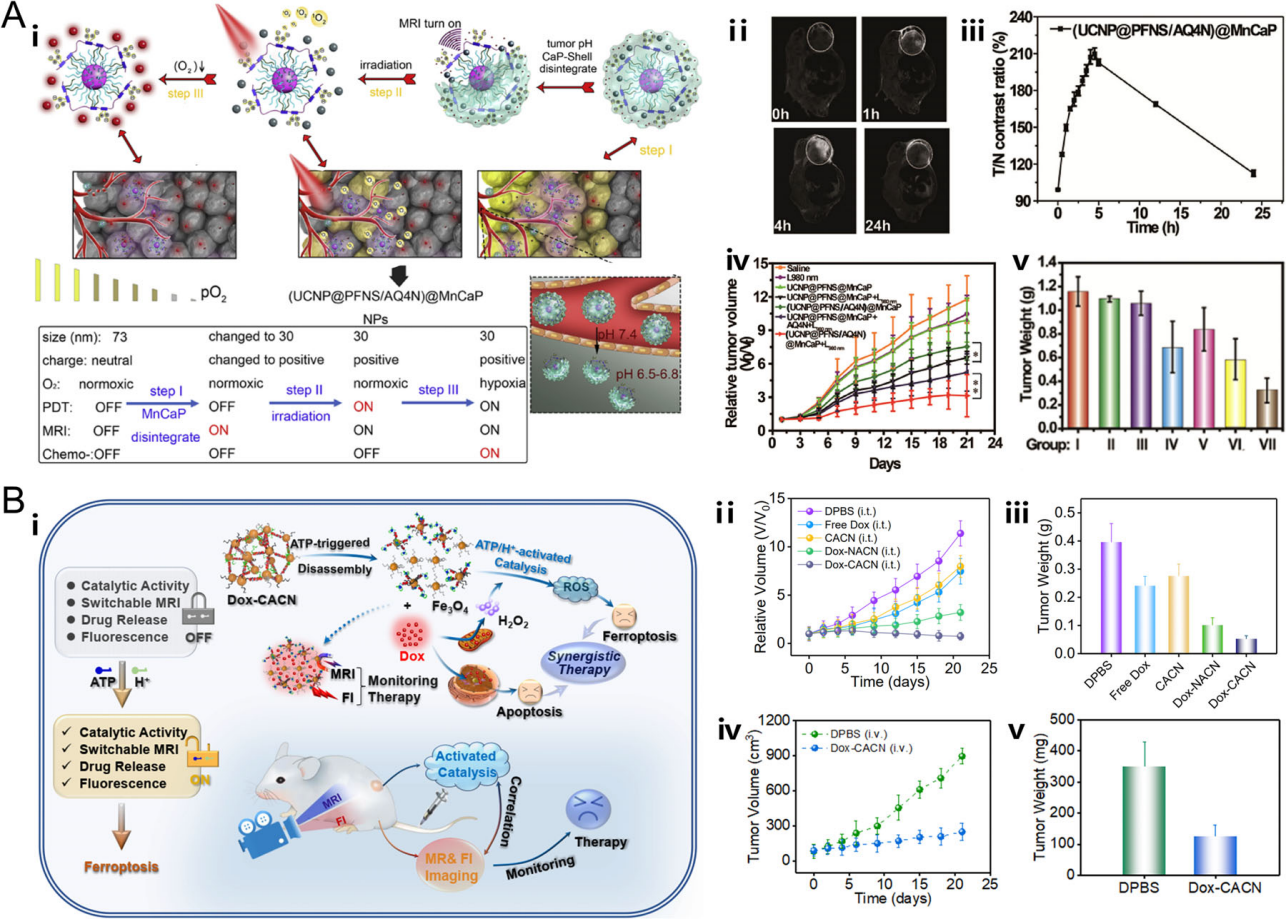
pH‐responsive nanoplatforms for combined therapy. (A) Chemotherapy and photodynamic therapy (PDT). The nanoplatform ((UCNP@PFNS/AQ4N)@MnCaP) is based on a core of PFNS‐modified up‐conversion nanoparticles (UCNPs) and a shell of Mn–doped Ca_3_(PO_4_)_2_. Schematic representation of (UCNP@PFNS/AQ4N)@MnCaP for synergetic PDT and chemotherapy under magnetic resonance imaging (MRI) guidance. The MRI and therapeutic outcomes of (UCNP@ PFNS/AQ4N)@MnCaP. Reproduced with permission.^[^ ^111^ ^]^ Copyright 2019, Elsevier Ltd. (B) Chemotherapy and chemodynamic therapy. The nanoplatform (Dox‐CACN) is based on DOX‐loaded iron oxide nanoclusters via DNA‐programmed self‐assembly. Schematic representation of dual key co‐activated nanoplatform for switchable MRI monitoring ferroptosis‐based synergistic therapy. The therapeutic outcomes of Dox‐CACN and other groups. Reproduced with permission.^[^ ^128^ ^]^ Copyright 2022, Elsevier Inc.

Despite the cooperative therapy of PDT and chemotherapy being much more effective for cancer therapy than monotherapies, premature release of anticancer drugs and hypoxia in the TME may lead to systemic toxicity and inferior therapeutic effects.^[^ ^193^ ^]^ To address these problems, Meng et al. developed an intelligent nanoflower composite with multistage pH/H_2_O_2_/GSH‐responsive characteristics which fully exploited TME features to achieve the controlled release of anticancer drugs into tumor sites and maximum synergistic curative effects. The inner polyphosphazene containing (bis‐(4‐hydroxyphenyl)‐disulfide) is pH‐ and GSH‐responsively biodegradable to unload the chemotherapeutic drug curcumin (CUR) and photosensitizer Ce6 in the TME. With MnO_2_ nanosheets as the outer shell, O_2_ concentration could be elevated by catalytic decomposition of H_2_O_2_ and Mn^2+^ ions could be released for T_1_‐MRI in the presence of acid and GSH. Therefore, with specific release of drugs, the relief of hypoxia, and the consumption of antioxidant, this TME‐responsive cancer theranostic nanoplatform achieved significant optimization of chemotherapy and PDT under the guidance of MRI.^[^ ^158^ ^]^ Instead of overcoming tumor hypoxia, another attractive strategy that utilizes the unfavorable PDT‐induced hypoxia to activate prodrug for supplementary chemotherapy has been proposed.^[^ ^194^ ^]^ Following this strategy, Fan and co‐workers developed the tandem activated theranostic nanoplatform (denoted as (UCNP@PFNS/AQ4N)@MnCaP) which utilized semiconducting polyelectrolyte‐based zwitterionic photosensitizer (PFNS) modified UCNPs as the core and pH‐sensitive Ca_3_(PO_4_)_2_ as the shell. Notably, this nanoplatform could realize different tumor distributions of photosensitizer (PFNS) and hypoxia‐activated prodrug (AQ4N) for amplified combination therapy. Furthermore, due to the existence of the pH‐responsive outer shell, the doped Mn^2+^ ions and incorporated prodrugs would be specifically released under acidic conditions of the tumor, thus achieving the pH‐activatable T_1_‐MRI and improving the precision of the combined therapy (Figure 9A).^[^ ^111^ ^]^

### 5.4 Chemotherapy/chemodynamic therapy

To integrate chemotherapy and chemodynamic therapy into a single system, Zhu et al. constructed bufalin‐loaded pH‐responsive hollow MnO_2_ NPs for chemo‐chemodynamic synergistic therapy under the guidance of MRI.^[^ ^90^ ^]^ Additionally, in a recent work of our group, we developed the DOX‐loaded iron oxide nanoclusters (denoted as Dox‐CACN) via DNA‐programmed self‐assembly. It is noteworthy that only upon co‐activation by dual stimuli of adenosine triphosphate (ATP) and acid in TME, the catalytic activity of the nanoplatform would be upregulated for specific CDT. Besides, ATP‐triggered disassembly of nanoclusters under acidic conditions resulted in the release of DOX and switchable T_2_/T_1_ MRI, which was correlated with ROS generation and Dox release. As a result, both intratumorally (i.t.) and intravenously (i.v.) injected Dox‐CACN markedly suppressed tumor development and minimized unwanted side effects through the co‐activation strategy and the guidance of switchable MRI (Figure 9B).^[^ ^128^ ^]^

### 5.5 Chemodynamic therapy/limotherapy

**FIGURE 10:**
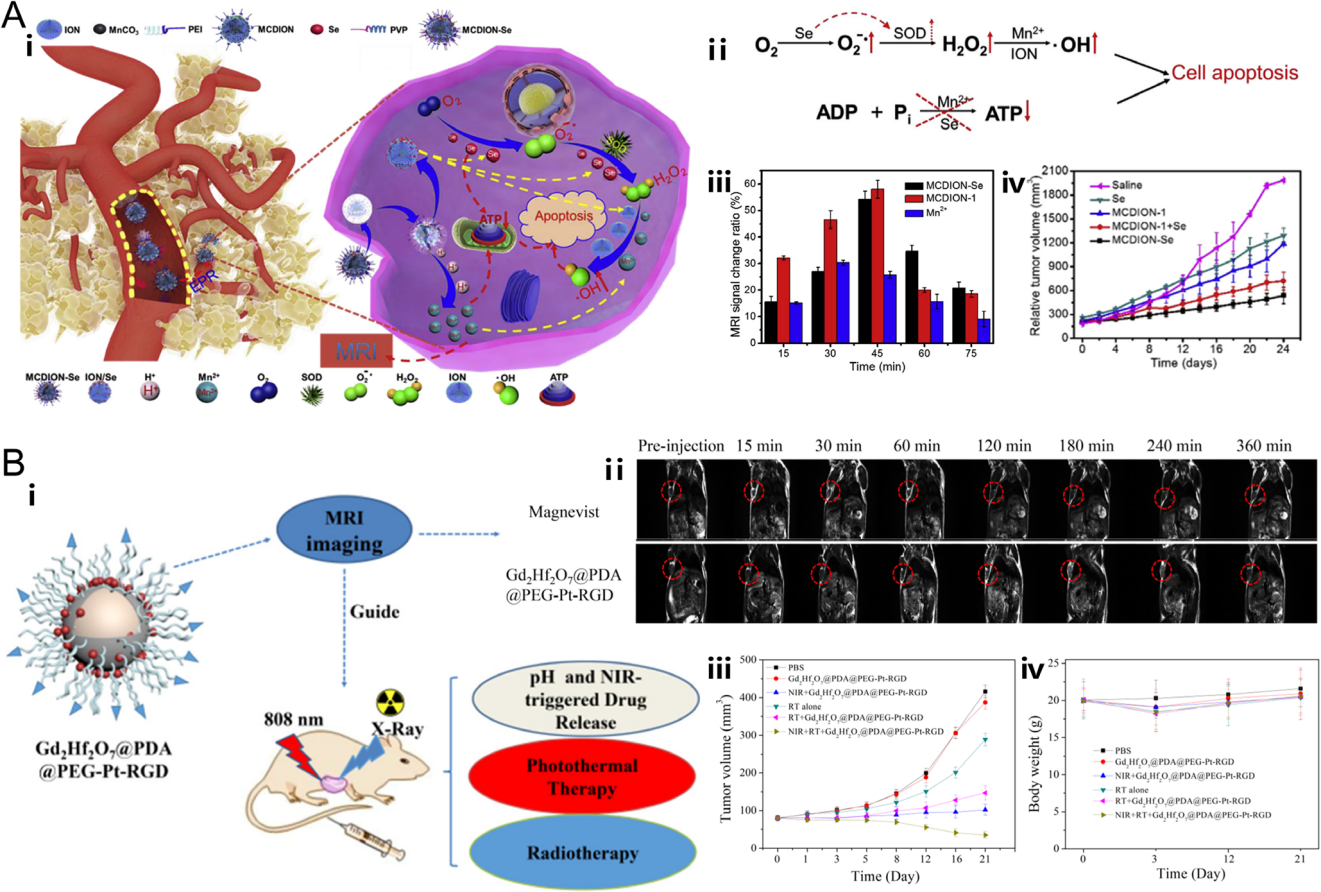
pH‐responsive nanoplatforms for combined therapy. (A) Chemodynamic therapy and limotherapy. The nanoplatform (MCDION‐Se) is based on nanoselenium (nano‐Se)‐coated MnCO_3_‐deposited nanoparticles. Schematic illustration of the cascade reaction of MCDION‐Se in the intracellular environment. The magnetic resonance imaging (MRI) and therapeutic outcomes of MCDION‐Se and other groups. Reproduced with permission.^[^ ^195^ ^]^ Copyright 2019, Elsevier Ltd. (B) Chemotherapy, radiotherapy, and photothermal therapy. The nanoplatform (Gd_2_Hf_2_O_7_@PDA@PEG‐Pt‐RGD) is based on polydopamine (PDA)‐modified and cisplatin‐loaded Gd_2_Hf_2_O_7_ NPs. Schematic illustration of Gd_2_Hf_2_O_7_@PDA@PEG‐Pt‐RGD for MRI‐guided synergistic therapy. The MRI and therapeutic outcomes of Gd_2_Hf_2_O_7_@PDA@PEG‐Pt‐RGD and other groups. Reproduced with permission.^[^ ^129^ ^]^ Copyright 2020, American Chemical Society.

A multifunctional theranostic nanoplatform (denoted as MCDION‐Se) combing CDT and limotherapy under the guidance of MRI was developed on the basis of nanoselenium (nano‐Se)‐coated MnCO_3_‐deposited nanoparticles.^[^ ^195^ ^]^ The pH‐dependent decomposition of MnCO_3_ would cause abundant release of Mn^2+^ ions which could catalyze H_2_O_2_ into hydroxyl radicals (·OH) through the Fenton‐like reaction, efficiently inducing apoptosis of the tumor cells. Furthermore, the pH‐dependent release of Mn^2+^ ions could produce enhanced T_1_ MR signal contrast under acidic conditions. Besides, nano‐Se could dramatically activate superoxide dismutase (SOD) and promote the production of superoxide anion radicals (SOARs) in tumor tissues. Subsequently, a large number of H_2_O_2_ was generated through SOD catalysis of SOARs, further improving CDT efficacy. In the meantime, the nano‐Se and Mn^2+^ could inhibit the production of ATP, thus starving tumor cells. As can be seen, the MCDION‐Se exhibited the best tumor inhibition efficiency and relatively high MRI signal change ratio 45 min after administration (Figure 10A).^[^ ^195^ ^]^

### 5.6 Chemotherapy/radiotherapy/PTT

In a radiotherapy (RT) process, X‐ray or γ‐ray is employed to activate water molecules to generate ROS, which can cause oxidative damage to proteins, lipids as well as DNAs and subsequently induce cell apoptosis.^[^ ^196^ ^]^ Nevertheless, RT has several drawbacks such as unclear side effects, resistance of radiation, and variation of radiation sensitivity for different individuals.^[^ ^196^ ^]^ In addition, the multiple drug resistance of the tumor could arise from the long‐term chemotherapy, which greatly impedes the curative effects.^[^ ^197^ ^]^ Fortunately, PTT is in the position to reduce drug resistance via hindering DNA repair, disrupting signal transduction, and denaturing cytoplasmic protein, which exhibits significant synergism with chemotherapy and radiotherapy.^[^ ^197^, ^198^ ^]^ Consequently, combining these three treatment modalities with MRI guidance can significantly enhance therapeutic efficacy. Recently, Pei et al. designed the PDA‐modified and cisplatin‐loaded Gd_2_Hf_2_O_7_ NPs (denoted as Gd_2_Hf_2_O_7_@PDA@PEG‐Pt‐RGD) which possess pH and NIR dual‐stimuli responsiveness for cisplatin release, strong NIR absorption and high X‐ray attenuation efficiency for combined chemo‐/photothermal‐/radiotherapy. Moreover, due to their ultrasmall size and surface modified by PDA and polyacrylic acid, the Gd_2_Hf_2_O_7_ NPs exhibited improved T_1_ MRI contrast ability. As can be seen, the Gd_2_Hf_2_O_7_@PDA@PEG‐Pt‐RGD has a much longer blood circulation time than Magnevist. Besides, when NIR and RT were simultaneously applied, the NIR+RT+Gd_2_Hf_2_O_7_@PDA@PEG‐Pt‐RGD would exhibit significant tumor suppression efficiency with no obvious side effects (Figure 10B).^[^ ^129^ ^]^ Additionally, a cisplatin‐loaded, PDA‐coated multi‐functional theranostic system was also developed for MRI‐guided combined therapy due to the simultaneous chemotherapeutic effect and radiosensitization of cisplatin and photothermal effect of PDA.^[^ ^40^ ^]^

## 6 CONCLUSION AND PROSPECTS

The acidic characteristic of the tumor site provides more motivation for tumor growth, invasion, and metastasis, but also offers a series of opportunities for cancer‐specific theranostic strategies. In this review, we summarized the pH‐responsive theranostic nanoplatforms with simultaneous diagnostic and therapeutic abilities. These nanoplatforms have drawn much attention in the field of nanomedicine because they provided great opportunity in the combat with cancer. On account of passive and active tumor targeting, theranostic nanoplatforms with pH‐responsiveness are able to deliver MRI contrast agents and therapeutic agents efficiently to the malignant tumor sites for MRI‐guided cancer therapy. The different design strategies applied to construct pH‐responsive theranostic nanoplatforms are presented in Table 1 (See Scheme 1).

**TABLE 1:** Summary of pH‐responsive components, types of MRI CAs, longitudinal and transverse relaxivity, and therapy modes of different pH‐responsive theranostic nanoplatforms discussed in the text.

pH‐responsive nanoplatform	pH‐responsive component	Type of MRI CAs	*r*_1_ or *r*_2_ [mM^−1^ s^−1^]	Therapy	Ref.
PEG‐GMF‐PPy NP	Tertiary amines	T_1_ (Gd^3+^)	*r*_1_ = 3.99 (pH 7.4, 7 T) *r*_1_ = 11.86 (pH 6.5, 7 T)	PTT	[31]
UCNPs@Au‐DOX	Hydrazone bonds	T_1_ (Gd^3+^)	*r*_1_ = 3.0595 (3 T)	PTT, pH‐activated CT	[43]
γ‐SD/PLL	Primary amines	T_2_ (γ‐Fe_2_O_3_)	*r*_2_ = 269.3 (1.5 T)	MH, pH‐activated CT	[192]
PMP‐CMD NP	Boronate esters	T_1_ (Mn^2+^)	*r*_1_ = 1.9 (pH 7.4, 0.5 T) *r*_1_ = 4.8 (pH 6.0, 0.5 T)	PDT	[54]
pH‐DEN	Imidazole groups	T_2_ (SPIONs)	*r*_2_ = 1595 (7 T)	pH‐activated CT	[60]
MEMSN	Acetals	T_1_ (Gd^3+^)	*r*_1_ = 6.9 (1.5 T)	pH‐activated CT	[67]
USIO NCs/DOX@CM	Benzoic imine bonds	T_1_ (USIO) T_2_ (USIO NCs)	*r*_1_ = 0.6 (pH 7.4, 0.5 T) *r*_1_ = 1.0 (pH 5.5, 0.5 T) *r*_2_ = 4.7 (pH 7.4, 0.5 T) *r*_2_ = 1.9 (pH 5.5, 0.5 T)	pH‐activated CT	[161]
FePt@FeO*_x_* @TAM‐PEG	Tertiary amines	T_2_ (FeO*_x_*)	*r*_2_ = 5.13 (7 T)	pH‐activated CDT	[181]
NC‐aP	Benzoic imine bonds	T_2_ (Fe_3_O_4_)	*r*_2_ = 178.8 (7 T)	Immunotherapy	[184]
UCNP@GA‐Fe	GA‐Fe(III)	T_1_ (Fe^3+^)	*r*_1_ = 3.2 (pH 7.0, 3 T) *r*_1_ = 9.2 (pH 4.0, 3 T)	PTT, pH‐activated CDT	[71]
Mn‐APPMSF	Fe(III)‐AQ4N	T_1_ (Mn^2+^)	*r*_1_ = 4.61 (9.4 T)	PTT, pH‐activated CT	[190]
CS‐MOFs@AS	MIL‐100(Fe)	T_1_ (Mn^2+^) T_2_ (Co, Fe)	*r*_1_ = 6.607 (0.5 T) *r*_2_ = 76.24 (0.5 T)	pH‐activated CT	[77]
MTX‐Mn@PEG	MTX‐Mn	T_1_ (Mn^2+^)	*r*_1_ = 7.76 (0.5 T)	pH‐activated CT	[85]
USMO@MSN	MnO*_x_*	T_1_ (Mn^2+^)	*r*_1_ = 0.68 (pH 7.4, 9.4 T) *r*_1_ = 5.61 (pH 4.5, 9.4 T)	pH‐activated CT	[94]
HMCNs	MnO*_x_*	T_1_ (Mn^2+^)	*r*_1_ = 0.79 (pH 7.4, 3 T) *r*_1_ = 8.81 (pH 5.0, 3 T)	pH‐activated CT	[95]
MnO*_x_*/Ti_3_C_2_‐SP	MnO*_x_*	T_1_ (Mn^2+^)	*r*_1_ = 1.47 (pH 7.4, 3 T) *r*_1_ = 8.09 (pH 6.0, 3 T)	PTT	[135]
Fe_3_O_4_/MnO*_x_*‐GO	MnO*_x_*	T_1_ (Mn^2+^) T_2_ (Fe_3_O_4_)	*r*_1_ = 1.2 (pH 7.4, 3 T) *r*_1_ = 5.6 (pH 6.0, 3 T) *r*_2_ = 65.9 (pH 7.4, 3 T) *r*_2_ = 103.17 (pH 6.0, 3 T)	MH, pH‐activated CT	[29]
Gd_2_O_3_ NSs	Gd_2_O_3_	T_1_ (Gd^3+^)	*r*_1_ = 6.732 (pH 7.4, 0.5 T) *r*_1_ = 7.301 (pH 5.0, 0.5 T)	pH‐activated CT	[97]
FA‐PYFGN‐CDDP	Gd_2_O_3_	T_1_ (Gd^3+^) T_2_ (SPIONs)	*r*_1_ = 7.91 (9.4 T) *r*_2_ = 386.5 (9.4 T)	pH‐activated CT	[156]
Fe_5_C_2_@Fe_3_O_4_ NPs	Fe_3_O_4_	T_1_ (Fe ions) T_2_ (Fe_3_O_4_)	*r*_1_ = 0.26 (pH 7.4, 3 T) *r*_1_ = 1.01 (pH 5.4, 3 T) *r*_2_ = 203.83 (pH 7.4, 3 T) *r*_2_ = 64.18 (pH 5.4, 3 T)	pH‐activated CDT	[99]
MCDION‐Se	MnCO_3_	T_1_ (Mn^2+^)	*r*_1_ = 2.7 (pH 7.4, 9.4 T) *r*_1_ = 7.1 (pH 5.5, 9.4 T)	Limotherapy, pH‐activated CDT	[195]
MnAsO*_x_* @SiO_2_	MnAsO*_x_*	T_1_ (Mn^2+^)	*r*_1_ = 4.8 (pH 7.4, 0.5 T) *r_1_* = 12.0 (pH 5.4, 0.5 T)	pH‐activated CT	[120]
CaCO_3_@Ce6(Mn)/DOX‐PEG	CaCO_3_	T_1_ (Mn^2+^)	*r_1_* = 1.156 (pH 7.4, 3 T) *r*_1_ = 11.48 (pH 5.5, 3 T)	pH‐activated PDT, pH‐activated CT	[100]
(UCNP@PFNS/AQ4N)@MnCaP	CaP	T_1_ (Mn^2+^)	*r*_1_ = 1.2 (pH 7.4, 7 T) *r*_1_ = 6.9 (pH 5.0, 7 T)	CT, PDT	[111]

*Note*: Other abbreviations can be found in the main text.

Abbreviations: MRI, magnetic resonance imaging; UCNP, up‐conversion nanoparticles; PTT, photothermal therapy; MH, magnetic hyperthermia; PDT, photodynamic therapy; CT, chemotherapy; HMCNs, hybrid mesoporous composite nanocapsules.

While significant progress has been made in the development of MRI‐based theranostic nanoplatforms which exhibited superb properties and undeniable potential in the growing era of personalized medicine, several challenges and obstacles still hinder the transition from bench to bedside, with few nanoplatforms undergoing clinical trials. One challenge in the clinical application of theranostic agents is the demand for high doses to achieve the desired diagnostic and therapeutic effects, which may bring unsafe and toxic problems. Besides, the multifunctional theranostic nanoplatforms typically require complicated synthesis processes and may cause premature release of cargo, which can lead to unwanted damage to healthy tissues. In addition, with the single stimulus response of merely low pH, the precision and specificity of the nanoplatform for theranostics could be undermined due to the existence of acidic compartments (e.g., lysosome, endosome) in normal cells. Consequently, it is appealing to design and develop dual/multiple stimuli‐responsive (e.g., hypoxia, acidosis, redox, ATP) nanoplatforms to realize more precise and specific diagnosis and therapy of tumors with minimized unwanted damage to the healthy tissues.

The incorporation of MRI as a diagnostic method has brought great convenience for the treatment of cancer. However, the MRI outcomes and the therapeutic effects seem to be cut apart with unclear correlation between them. Therefore, it is appealing to design and develop a theranostic nanoplatform in which the MRI signal intensities could reflect the therapeutic effects in real time with a direct correlation. Furthermore, the biosafety of the MRI CAs remains a big concern. For instance, exposure to Gd‐containing CAs was associated with the development of nephrogenic systemic fibrosis in patients with renal insufficiency.^[^ ^199^ ^]^ Although Mn‐based CAs possess improved biocompatibility over Gd‐based CAs, the toxicity problem still existed.^[^ ^136^ ^]^ Compared to Mn^2+^, Fe^3+^ is considered more biocompatible because the human body is capable of metabolizing iron ions, an innate element of hemoglobin. Thus, we assume that Fe‐based CAs might be a more suitable candidate for further clinical use. Besides, for the next generation of pH‐responsive MRI CAs, it would be beneficial to design smart MRI CAs, which could produce a larger variation of signal intensity in response to a more subtle pH gradient. In this case, it might be able to realize the precise imaging of subcellular structures which possess the different ranges of pH variation. In addition, single MRI modality is limited by its poor sensitivity. Thus, it can be combined with other imaging modalities (e.g., optical imaging, positron emission tomography, or single‐photon emission computed tomography) which possess the desired sensitivity to obtain the imaging results with high accuracy.

In the end, translating basic research into clinical use requires the demonstration of improved therapeutic efficacy over the current therapies and sufficient biocompatibility. There is increasing evidence that the synergistic strategy combining different drugs and mechanisms with improved curative effects is extremely appealing to combat the heterogeneous and multi‐drug resistant tumors. Consequently, the multifunctional theranostic nanoplatforms which exhibited considerable values for biomedical applications due to their superior properties and potential for clinical use, are well suited for this purpose. We believe that, with the constant optimization of pH‐responsive theranostic nanoplatforms, the diagnostic and therapeutic capabilities would be improved while the side effects produced during the treatment procedure would be minimized as much as possible. Although breakthroughs in this area largely rely on the collaboration between different fields of knowledge, the future of theranostic nanoplatform is promising and we anticipate that more attempts based on this would be made in the near future.

## CONFLICT OF INTEREST STATEMENT

The authors declare no conflict of interest.

## References

[1] L. Cheng , C. Wang , L. Feng , K. Yang , Z. Liu , Chem. Rev. 2014, 114, 10869. 25260098 10.1021/cr400532z

[2] L. Zhu , D. Wang , X. Wei , X. Zhu , J. Li , C. Tu , Y. Su , J. Wu , B. Zhu , D. Yan , J. Controlled Release 2013, 169, 228. 10.1016/j.jconrel.2013.02.015 23485450

[3] S. Zhang , X. Qian , L. Zhang , W. Peng , Y. Chen , Nanoscale 2015, 7, 7632. 25785502 10.1039/c5nr00451a

[4] L. Zhang , Z. Yang , W. Zhu , Z. Ye , Y. Yu , Z. Xu , J. Ren , P. Li , ACS Biomater. Sci. Eng. 2017, 3, 1690. 33429651 10.1021/acsbiomaterials.7b00204

[5] V. Estrella , T. Chen , M. Lloyd , J. Wojtkowiak , H. H. Cornnell , A. Ibrahim‐Hashim , K. Bailey , Y. Balagurunathan , J. M. Rothberg , B. F. Sloane , J. Johnson , R. A. Gatenby , R. J. Gillies , Cancer Res. 2013, 73, 1524. 23288510 10.1158/0008-5472.CAN-12-2796 PMC3594450

[6] D. Ribatti , Tumor Microenvironment Regulation of Tumor Expansion, 1st ed., Academic Press, London 2021.

[7] S. H. Crayton , A. Tsourkas , ACS Nano 2011, 5, 9592. 22035454 10.1021/nn202863x PMC3246562

[8] Z. Ge , S. Liu , Chem. Soc. Rev. 2013, 42, 7289. 23549663 10.1039/c3cs60048c

[9] F. Visioli , Y. Wang , G. N. Alam , Y. Ning , P. V. Rados , J. E. Nor , P. J. Polverini , PLoS One 2014, 9, e101053. 24964091 10.1371/journal.pone.0101053 PMC4071032

[10] Z. Yang , N. Sun , R. Cheng , C. Zhao , Z. Liu , X. Li , J. Liu , Z. Tian , Biomaterials 2017, 147, 53. 28930649 10.1016/j.biomaterials.2017.09.013

[11] X. Zhang , S. Wang , G. Cheng , P. Yu , J. Chang , X. Chen , Matter 2021, 4, 26. 33718863 10.1016/j.matt.2020.10.002 PMC7945719

[12] Y. Wang , S. Song , J. Liu , D. Liu , H. Zhang , Angew. Chem., Int. Ed. 2015, 54, 536. 10.1002/anie.201409519 25366670

[13] L. Teng , G. Song , Y. Liu , X. Han , Z. Li , Y. Wang , S. Huan , X. B. Zhang , W. Tan , J. Am. Chem. Soc. 2019, 141, 13572. 31370392 10.1021/jacs.9b05901

[14] H. Yuan , Y. Zhao , C. Yang , C. Zhang , Y. Yang , H. Meng , S. Huan , G. Song , X. Zhang , Sci. China: Chem. 2020, 63, 924.

[15] W. H. Chiang , W. C. Huang , C. W. Chang , M. Y. Shen , Z. F. Shih , Y. F. Huang , S. C. Lin , H. C. Chiu , J. Controlled Release 2013, 168, 280. 10.1016/j.jconrel.2013.03.029 23562635

[16] S. Ray , Z. Li , C. H. Hsu , L. P. Hwang , Y. C. Lin , P. T. Chou , Y. Y. Lin , Theranostics 2018, 8, 6322. 30613300 10.7150/thno.27828 PMC6299700

[17] H. Liu , C. Lu , L. Han , X. Zhang , G. Song , Coord. Chem. Rev. 2021, 441, 213978.

[18] L. Teng , X. Han , Y. Liu , C. Lu , B. Yin , S. Huan , X. Yin , X. B. Zhang , G. Song , Angew. Chem., Int. Ed. 2021, 60, 26142. 10.1002/anie.202110427 34554633

[19] L. Wu , Y. Ishigaki , W. Zeng , T. Harimoto , B. Yin , Y. Chen , S. Liao , Y. Liu , Y. Sun , X. Zhang , Y. Liu , Y. Liang , P. Sun , T. Suzuki , G. Song , Q. Fan , D. Ye , Nat. Commun. 2021, 12, 6145. 34686685 10.1038/s41467-021-26380-y PMC8536768

[20] Y. Ma , L. Xu , B. Yin , J. Shang , F. Chen , J. Xu , Z. L. Song , B. Nan , G. Song , X. B. Zhang , Nano Lett. 2021, 21, 4484. 33978427 10.1021/acs.nanolett.1c01359

[21] B. Brito , T. W. Price , J. Gallo , M. Banobre‐Lopez , G. J. Stasiuk , Theranostics 2021, 11, 8706. 34522208 10.7150/thno.57004 PMC8419031

[22] C. Lu , L. Han , J. Wang , J. Wan , G. Song , J. Rao , Chem. Soc. Rev. 2021, 50, 8102. 34047311 10.1039/d0cs00260g

[23] K. S. Kim , W. Park , J. Hu , Y. H. Bae , K. Na , Biomaterials 2014, 35, 337. 24139764 10.1016/j.biomaterials.2013.10.004

[24] L. Zhu , Y. Yang , K. Farquhar , J. Wang , C. Tian , J. Ranville , S. G. Boyes , ACS Appl. Mater. Interfaces 2016, 8, 5040. 26790986 10.1021/acsami.5b12463

[25] F. Mouffouk , T. Simao , D. F. Dornelles , A. D. Lopes , P. Sau , J. Martins , K. M. Abu‐Salah , S. A. Alrokayan , A. M. Rosa da Costa , N. R. dos Santos , Int. J. Nanomed. 2015, 10, 63. 10.2147/IJN.S71190 PMC4275056 25565804

[26] J. Hu , T. Liu , G. Zhang , F. Jin , S. Liu , Macromol. Rapid Commun. 2013, 34, 749. 23193017 10.1002/marc.201200613

[27] J. H. Park , H. J. Cho , H. Y. Yoon , I. S. Yoon , S. H. Ko , J. S. Shim , J. H. Cho , J. H. Park , K. Kim , I. C. Kwon , D. D. Kim , J. Controlled Release 2014, 174, 98. 10.1016/j.jconrel.2013.11.016 24280260

[28] G. Zhang , R. Du , J. Qian , X. Zheng , X. Tian , D. Cai , J. He , Y. Wu , W. Huang , Y. Wang , X. Zhang , K. Zhong , D. Zou , Z. Wu , Nanoscale 2017, 10, 488. 29231948 10.1039/c7nr07957e

[29] Y. Chen , P. Xu , Z. Shu , M. Wu , L. Wang , S. Zhang , Y. Zheng , H. Chen , J. Wang , Y. Li , J. Shi , Adv. Funct. Mater. 2014, 24, 4386.

[30] C. W. Lin , S. J. Tseng , I. M. Kempson , S. C. Yang , T. M. Hong , P. C. Yang , Biomaterials 2013, 34, 4387. 23478033 10.1016/j.biomaterials.2013.02.058

[31] S. Wang , Z. Zhou , G. Yu , N. Lu , Y. Liu , Y. Dai , X. Fu , J. Wang , X. Chen , ACS Appl. Mater. Interfaces 2018, 10, 28382. 30085649 10.1021/acsami.8b09670

[32] J. Chen , H. Chen , P. Li , H. Diao , S. Zhu , L. Dong , R. Wang , T. Guo , J. Zhao , J. Zhang , Biomaterials 2011, 32, 4793. 21489619 10.1016/j.biomaterials.2011.03.041

[33] G. H. Gao , J. W. Lee , M. K. Nguyen , G. H. Im , J. Yang , H. Heo , P. Jeon , T. G. Park , J. H. Lee , D. S. Lee , J. Controlled Release 2011, 155, 11. 10.1016/j.jconrel.2010.09.012 20854855

[34] H. Ma , Y. Liu , M. Shi , X. Shao , W. Zhong , W. Liao , M. M. Xing , Biomacromolecules 2015, 16, 4022. 26477267 10.1021/acs.biomac.5b01039

[35] Y. F. Zeng , S. J. Tseng , I. M. Kempson , S. F. Peng , W. T. Wu , J. R. Liu , Biomaterials 2012, 33, 9239. 23026709 10.1016/j.biomaterials.2012.09.018

[36] X. Kang , D. Yang , Y. Dai , M. Shang , Z. Cheng , X. Zhang , H. Lian , P. Ma , J. Lin , Nanoscale 2013, 5, 253. 23154448 10.1039/c2nr33130f

[37] H. Ren , L. Zhang , J. An , T. Wang , L. Li , X. Si , L. He , X. Wu , C. Wang , Z. Su , ChemComm 2014, 50, 1000. 10.1039/c3cc47666a 24306285

[38] H. Shi , L. Li , L. Zhang , T. Wang , C. Wang , D. Zhu , Z. Su , CrystEngComm 2015, 17, 4768.

[39] J. Zhao , Z. Zhang , X. Wang , J. Alloys Compd. 2020, 820, 153142.

[40] C. Yang , X. Mi , H. Su , J. Yang , Y. Gu , L. Zhang , W. Sun , X. Liang , C. Zhang , Biomater. Sci. 2019, 7, 2076. 30860522 10.1039/c8bm01492b

[41] G. Zhang , J. Gao , J. Qian , L. Zhang , K. Zheng , K. Zhong , D. Cai , X. Zhang , Z. Wu , ACS Appl. Mater. Interfaces 2015, 7, 14192. 26084052 10.1021/acsami.5b04294

[42] N. Wang , C. Liu , W. Yao , H. Zhou , S. Yu , H. Chen , W. Qiao , Colloids Surf., B 2021, 205, 111866. 10.1016/j.colsurfb.2021.111866 34044333

[43] R. Wei , W. Xi , H. Wang , J. Liu , T. Mayr , L. Shi , L. Sun , Nanoscale 2017, 9, 12885. 28650053 10.1039/c7nr02280h

[44] S. H. Hosseini , N. Zohreh , N. Karimi , N. Gaeini , S. Alipour , F. Seidi , N. Gholipour , Int. J. Biol. Macromol. 2020, 158, 994. 32434748 10.1016/j.ijbiomac.2020.05.040

[45] X. Ding , Y. Liu , J. Li , Z. Luo , Y. Hu , B. Zhang , J. Liu , J. Zhou , K. Cai , ACS Appl. Mater. Interfaces 2014, 6, 7395. 24749476 10.1021/am500818m

[46] Y. Chang , N. Liu , L. Chen , X. Meng , Y. Liu , Y. Li , J. Wang , J. Mater. Chem. 2012, 22, 9594.

[47] X. Q. Yang , J. J. Grailer , I. J. Rowland , A. Javadi , S. A. Hurley , V. Z. Matson , D. A. Steeber , S. Gong , ACS Nano 2010, 4, 6805. 20958084 10.1021/nn101670k

[48] A. Bernkop‐Schnurch , S. Dunnhaupt , Eur. J. Pharm. Biopharm. 2012, 81, 463. 22561955 10.1016/j.ejpb.2012.04.007

[49] F. Croisier , C. Jérôme , Eur. Polym. J. 2013, 49, 780.

[50] S. Y. Yun , D. Seo , H.‐J. Kim , D.‐G. Jeung , Y. K. Jeong , J.‐M. Oh , J. K. Park , J. Ind. Eng. Chem. 2021, 95, 28.

[51] A. Shanavas , S. Sasidharan , D. Bahadur , R. Srivastava , J. Colloid Interface Sci. 2017, 486, 112. 27697648 10.1016/j.jcis.2016.09.060

[52] C. Lin , K. Sun , C. Zhang , T. Tan , M. Xu , Y. Liu , C. Xu , Y. Wang , L. Li , A. Whittaker , Microporous Mesoporous Mater. 2020, 293, 109775.

[53] C. Wang , S. Ravi , U. S. Garapati , M. Das , M. Howell , J. MallelaMallela , S. Alwarapan , S. S. Mohapatra , S. Mohapatra , J. Mater. Chem. B 2013, 1, 4396. 24883188 10.1039/C3TB20452A PMC4036826

[54] Y. Hao , C. Zheng , L. Wang , J. Zhang , X. Niu , Q. Song , Q. Feng , H. Zhao , L. Li , H. Zhang , Z. Zhang , Y. Zhang , Acta Biomater. 2017, 62, 293. 28842332 10.1016/j.actbio.2017.08.028

[55] Y. K. Reshetnyak , O. A. Andreev , M. Segala , V. S. Markin , D. M. Engelman , Proc. Natl. Acad. Sci. U. S. A. 2008, 105, 15340. 18829441 10.1073/pnas.0804746105 PMC2556629

[56] O. A. Andreev , A. G. Karabadzhak , D. Weerakkody , G. O. Andreev , D. M. Engelman , Y. K. Reshetnyak , Proc. Natl. Acad. Sci. U. S. A. 2010, 107, 4081. 20160113 10.1073/pnas.0914330107 PMC2840156

[57] A. G. Pershina , O. Y. Brikunova , A. M. Demin , O. B. Shevelev , I. A. Razumov , E. L. Zavjalov , D. Malkeyeva , E. Kiseleva , N. V. Krakhmal , S. V. Vtorushin , V. L. Yarnykh , V. V. Ivanov , R. I. Pleshko , V. P. Krasnov , L. M. Ogorodova , Nanomedicine 2020, 23, 102086. 31449887 10.1016/j.nano.2019.102086

[58] Y. Wei , R. Liao , A. A. Mahmood , H. Xu , Q. Zhou , Acta Biomater. 2017, 55, 194. 28363789 10.1016/j.actbio.2017.03.046

[59] A. M. Demin , A. G. Pershina , A. S. Minin , O. Y. Brikunova , A. M. Murzakaev , N. A. Perekucha , A. V. Romashchenko , O. B. Shevelev , M. A. Uimin , I. V. Byzov , D. Malkeyeva , E. Kiseleva , L. V. Efimova , S. V. Vtorushin , L. M. Ogorodova , V. P. Krasnov , ACS Appl. Mater. Interfaces 2021, 13, 36800. 34324807 10.1021/acsami.1c07748

[60] W. Park , J. Chen , S. Cho , S. J. Park , A. C. Larson , K. Na , D. H. Kim , ACS Appl. Mater. Interfaces 2016, 8, 12711. 27159350 10.1021/acsami.6b03505 PMC4943858

[61] D. J. Lee , Y. T. Oh , E. S. Lee , Int. J. Pharm. 2014, 471, 127. 24858382 10.1016/j.ijpharm.2014.05.029

[62] X. C. Zheng , W. Ren , S. Zhang , T. Zhong , X. C. Duan , Y. F. Yin , M. Q. Xu , Y. L. Hao , Z. T. Li , H. Li , M. Liu , Z. Y. Li , X. Zhang , Int. J. Nanomed. 2018, 13, 1495. 10.2147/IJN.S157082 PMC5856286 29559778

[63] S. I. Park , E. O. Lee , J. W. Kim , Y. J. Kim , S. H. Han , J. D. Kim , J. Colloid Interface Sci. 2011, 364, 31. 21885053 10.1016/j.jcis.2011.07.046

[64] S. M. Lee , Y. Song , B. J. Hong , K. W. MacRenaris , D. J. Mastarone , T. V. O'Halloran , T. J. Meade , S. T. Nguyen , Angew. Chem., Int. Ed. 2010, 49, 9960. 10.1002/anie.201004867 PMC3096927 21082634

[65] Y. J. Tseng , S. W. Chou , J. J. Shyue , S. Y. Lin , J. K. Hsiao , P. T. Chou , ACS Nano 2016, 10, 5809. 27163375 10.1021/acsnano.5b08130

[66] J. Wu , Z. Meng , A. A. Exner , X. Cai , X. Xie , B. Hu , Y. Chen , Y. Zheng , Biomaterials 2021, 276, 121001. 34274775 10.1016/j.biomaterials.2021.121001

[67] Y. Chen , K. Ai , J. Liu , G. Sun , Q. Yin , L. Lu , Biomaterials 2015, 60, 111. 25988726 10.1016/j.biomaterials.2015.05.003

[68] F. Liu , X. He , H. Chen , J. Zhang , H. Zhang , Z. Wang , Nat. Commun. 2015, 6, 8003. 26245151 10.1038/ncomms9003 PMC4918390

[69] C. Zhang , J. Li , C. Yang , S. Gong , H. Jiang , M. Sun , C. Qian , Nanomedicine 2020, 23, 102071. 31442581 10.1016/j.nano.2019.102071

[70] C. Zhang , J. Li , C. Lu , T. Yang , Y. Zhao , L. Teng , Y. Yang , G. Song , X.‐B. Zhang , CCS Chem. 2021, 3, 2126.

[71] P. Zhang , Y. Hou , J. Zeng , Y. Li , Z. Wang , R. Zhu , T. Ma , M. Gao , Angew. Chem., Int. Ed. 2019, 58, 11088. 10.1002/anie.201904880 31131511

[72] W. Morris , W. E. Briley , E. Auyeung , M. D. Cabezas , C. A. Mirkin , J. Am. Chem. Soc. 2014, 136, 7261. 24818877 10.1021/ja503215w

[73] T. Baati , L. Njim , F. Neffati , A. Kerkeni , M. Bouttemi , R. Gref , M. F. Najjar , A. Zakhama , P. Couvreur , C. Serre , P. Horcajada , Chem. Sci. 2013, 4, 1597.

[74] Y. Liu , C. Zhang , H. Liu , Y. Li , Z. Xu , L. Li , A. Whittaker , J. Alloys Compd. 2018, 749, 939.

[75] Z. Wang , W. Yu , N. Yu , X. Li , Y. Feng , P. Geng , M. Wen , M. Li , H. Zhang , Z. Chen , Chem. Eng. J. 2020, 400, 125877.

[76] D. Wang , J. Zhou , R. Chen , R. Shi , G. Zhao , G. Xia , R. Li , Z. Liu , J. Tian , H. Wang , Z. Guo , H. Wang , Q. Chen , Biomaterials 2016, 100, 27. 27240160 10.1016/j.biomaterials.2016.05.027

[77] D. Wang , J. Zhou , R. Chen , R. Shi , C. Wang , J. Lu , G. Zhao , G. Xia , S. Zhou , Z. Liu , H. Wang , Z. Guo , Q. Chen , Chem. Mater. 2017, 29, 3477.

[78] K. Xin , M. Li , D. Lu , X. Meng , J. Deng , D. Kong , D. Ding , Z. Wang , Y. Zhao , ACS Appl. Mater. Interfaces 2017, 9, 80. 27957858 10.1021/acsami.6b09425

[79] F. Liu , X. He , J. Zhang , H. Chen , H. Zhang , Z. Wang , J. Mater. Chem. B 2015, 3, 6731. 32262465 10.1039/c5tb01159k

[80] G. Huang , R. Liu , Y. Hu , S.‐H. Li , Y. Wu , Y. Qiu , J. Li , H.‐H. Yang , Sci. China: Chem. 2018, 61, 806.

[81] G. Fan , F. Li , D. G. Evans , X. Duan , Chem. Soc. Rev. 2014, 43, 7040. 25001024 10.1039/c4cs00160e

[82] C. Zhang , L. Li , F. Y. Han , X. Yu , X. Tan , C. Fu , Z. P. Xu , A. K. Whittaker , Small 2019, 15, e1902309. 31328398 10.1002/smll.201902309

[83] B. Li , Z. Gu , N. Kurniawan , W. Chen , Z. P. Xu , Adv. Mater. 2017, 29, 1700373. 10.1002/adma.201700373 28585312

[84] H. Zuo , W. Chen , B. Li , K. Xu , H. Cooper , Z. Gu , Z. P. Xu , Chem. Eur. J. 2017, 23, 14299. 28762580 10.1002/chem.201702835

[85] Y. Wu , L. Xu , J. Qian , L. Shi , Y. Su , Y. Wang , D. Li , X. Zhu , Biomater. Sci. 2020, 8, 712. 31777869 10.1039/c9bm01584a

[86] C. Y. Sun , C. Qin , X. L. Wang , G. S. Yang , K. Z. Shao , Y. Q. Lan , Z. M. Su , P. Huang , C. G. Wang , E. B. Wang , Dalton Trans. 2012, 41, 6906. 22580798 10.1039/c2dt30357d

[87] M. He , J. Zhou , J. Chen , F. Zheng , D. Wang , R. Shi , Z. Guo , H. Wang , Q. Chen , J. Mater. Chem. B 2015, 3, 9033. 32263034 10.1039/c5tb01830g

[88] D. Wang , J. Zhou , R. Shi , H. Wu , R. Chen , B. Duan , G. Xia , P. Xu , H. Wang , S. Zhou , C. Wang , H. Wang , Z. Guo , Q. Chen , Theranostics 2017, 7, 4605. 29158848 10.7150/thno.20363 PMC5695152

[89] R. A. Y. Yao , X. Guo , W. Jiang , M. Jiang , J. Yang , Y. Li , O. O. Atinuke , X. Hu , Y. Li , X. Wang , L. Yang , X. Yang , K. Wang , J. Hu , X. Sun , ACS Appl. Mater. Interfaces 2021, 13, 18604. 33856200 10.1021/acsami.1c04310

[90] H. Wang , D. H. Bremner , K. Wu , X. Gong , Q. Fan , X. Xie , H. Zhang , J. Wu , L.‐M. Zhu , Chem. Eng. J. 2020, 382, 122848.

[91] Y. Chen , D. Ye , M. Wu , H. Chen , L. Zhang , J. Shi , L. Wang , Adv. Mater. 2014, 26, 7019. 25156250 10.1002/adma.201402572

[92] J. Shang , B. Xie , Y. Li , X. Wei , N. Du , H. Li , W. Hou , R. Zhang , ACS Nano 2016, 10, 5916. 27187574 10.1021/acsnano.6b01229

[93] T. He , C. Jiang , J. He , Y. Zhang , G. He , J. Wu , J. Lin , X. Zhou , P. Huang , Adv. Mater. 2021, 33, e2008540. 33645863 10.1002/adma.202008540

[94] D. Wang , H. Lin , G. Zhang , Y. Si , H. Yang , G. Bai , C. Yang , K. Zhong , D. Cai , Z. Wu , R. Wang , D. Zou , ACS Appl. Mater. Interfaces 2018, 10, 31114. 30141893 10.1021/acsami.8b11408

[95] Y. Chen , Q. Yin , X. Ji , S. Zhang , H. Chen , Y. Zheng , Y. Sun , H. Qu , Z. Wang , Y. Li , X. Wang , K. Zhang , L. Zhang , J. Shi , Biomaterials 2012, 33, 7126. 22789722 10.1016/j.biomaterials.2012.06.059

[96] Q. Sun , F. He , C. Sun , X. Wang , C. Li , J. Xu , D. Yang , H. Bi , S. Gai , P. Yang , ACS Appl. Mater. Interfaces 2018, 10, 33901. 30207691 10.1021/acsami.8b10207

[97] M. Wu , Y. Xue , N. Li , H. Zhao , B. Lei , M. Wang , J. Wang , M. Luo , C. Zhang , Y. Du , C. Yan , Angew. Chem., Int. Ed. 2019, 58, 6880. 10.1002/anie.201812972 30680857

[98] X. Li , X. Wang , A. Ito , ChemComm 2020, 56, 8186.

[99] J. Yu , F. Zhao , W. Gao , X. Yang , Y. Ju , L. Zhao , W. Guo , J. Xie , X. J. Liang , X. Tao , J. Li , Y. Ying , W. Li , J. Zheng , L. Qiao , S. Xiong , X. Mou , S. Che , Y. Hou , ACS Nano 2019, 13, 10002. 31433945 10.1021/acsnano.9b01740

[100] Z. Dong , L. Feng , W. Zhu , X. Sun , M. Gao , H. Zhao , Y. Chao , Z. Liu , Biomaterials 2016, 110, 60. 27710833 10.1016/j.biomaterials.2016.09.025

[101] S. Nakki , J. T. Wang , J. Wu , L. Fan , J. Rantanen , T. Nissinen , M. I. Kettunen , M. Backholm , R. H. A. Ras , K. T. Al‐Jamal , V. P. Lehto , W. Xu , Int. J. Pharm. 2019, 554, 327. 30391665 10.1016/j.ijpharm.2018.10.074

[102] Z. Yi , Z. Luo , N. D. Barth , X. Meng , H. Liu , W. Bu , A. All , M. Vendrell , X. Liu , Adv. Mater. 2019, 31, e1901851. 31364218 10.1002/adma.201901851

[103] X. Zhu , H. Xiong , Q. Zhou , Z. Zhao , Y. Zhang , Y. Li , S. Wang , S. Shi , ACS Appl. Mater. Interfaces 2021, 13, 18462. 33871955 10.1021/acsami.0c22624

[104] Y. Cheng , S. Zhang , N. Kang , J. Huang , X. Lv , K. Wen , S. Ye , Z. Chen , X. Zhou , L. Ren , ACS Appl. Mater. Interfaces 2017, 9, 19296. 28508635 10.1021/acsami.7b03087

[105] M. Gou , S. Li , L. Zhang , L. Li , C. Wang , Z. Su , ChemComm 2016, 52, 11068. 10.1039/c6cc05515j 27561158

[106] P. Mi , D. Kokuryo , H. Cabral , H. Wu , Y. Terada , T. Saga , I. Aoki , N. Nishiyama , K. Kataoka , Nat. Nanotechnol. 2016, 11, 724. 27183055 10.1038/nnano.2016.72

[107] L. H. Fu , Y. R. Hu , C. Qi , T. He , S. Jiang , C. Jiang , J. He , J. Qu , J. Lin , P. Huang , ACS Nano 2019, 13, 13985. 31833366 10.1021/acsnano.9b05836

[108] L. H. Fu , C. Qi , Y. R. Hu , J. Lin , P. Huang , Adv. Mater. 2019, 31, 1808325. 10.1002/adma.201808325 30907460

[109] L. H. Fu , C. Li , W. Yin , Y. R. Hu , T. Sun , Y. Wan , J. Lin , Z. Li , P. Huang , Adv. Healthcare Mater. 2021, 10, 2101563. 10.1002/adhm.202101563 34632723

[110] W. Fan , N. Lu , P. Huang , Y. Liu , Z. Yang , S. Wang , G. Yu , Y. Liu , J. Hu , Q. He , J. Qu , T. Wang , X. Chen , Angew. Chem., Int. Ed. 2017, 56, 1229. 10.1002/anie.201610682 27936311

[111] Y. Ji , F. Lu , W. Hu , H. Zhao , Y. Tang , B. Li , X. Hu , X. Li , X. Lu , Q. Fan , W. Huang , Biomaterials 2019, 219, 119393. 31382206 10.1016/j.biomaterials.2019.119393

[112] N. N. Zhang , R. S. Yu , M. Xu , X. Y. Cheng , C. M. Chen , X. L. Xu , C. Y. Lu , K. J. Lu , M. J. Chen , M. L. Zhu , Q. Y. Weng , J. G. Hui , Q. Zhang , Y. Z. Du , J. S. Ji , Nanomedicine 2018, 14, 2167. 30017962 10.1016/j.nano.2018.06.014

[113] J. Zhang , M. Jin , Y. I. Park , L. Jin , B. Quan , Ceram. Int. 2020, 46, 18632.

[114] X. Li , H. Zhou , Z. Niu , K. Zheng , D. Niu , W. Zhao , X. Liu , W. Si , C. Li , P. Wang , J. Cao , Y. Li , G. Wen , ACS Appl. Mater. Interfaces 2020, 12, 24644. 32407072 10.1021/acsami.0c07018

[115] J. Chen , W. J. Zhang , Z. Guo , H. B. Wang , D. D. Wang , J. J. Zhou , Q. W. Chen , ACS Appl. Mater. Interfaces 2015, 7, 5373. 25685956 10.1021/acsami.5b00727

[116] X. W. Li , W. R. Zhao , Y. J. Liu , X. H. Liu , P. Shi , Y. S. Li , J. L. Shi , J. Mater. Chem. B 2016, 4, 4313. 32263413 10.1039/c6tb00718j

[117] X. Li , W. Zhao , X. Liu , K. Chen , S. Zhu , P. Shi , Y. Chen , J. Shi , Acta Biomater. 2016, 30, 378. 26602820 10.1016/j.actbio.2015.11.036

[118] J. Chen , W. Zhang , M. Zhang , Z. Guo , H. Wang , M. He , P. Xu , J. Zhou , Z. Liu , Q. Chen , Nanoscale 2015, 7, 12542. 26140326 10.1039/c5nr02402a

[119] H. M. Chen , R. C. MacDonald , S. Y. Li , N. L. Krett , S. T. Rosen , T. V. O'Halloran , J. Am. Chem. Soc. 2006, 128, 13348. 17031934 10.1021/ja064864h

[120] Z. H. Zhao , X. M. Wang , Z. J. Zhang , H. Zhang , H. Y. Liu , X. L. Zhu , H. Li , X. Q. Chi , Z. Y. Yin , J. H. Gao , ACS Nano 2015, 9, 2749. 25688714 10.1021/nn506640h

[121] K. Zhang , H. Lin , J. Mao , X. Luo , R. Wei , Z. Su , B. Zhou , D. Li , J. Gao , H. Shan , Biomater. Sci. 2019, 7, 2480. 30957825 10.1039/c9bm00216b

[122] T. H. Shin , Y. Choi , S. Kim , J. Cheon , Chem. Soc. Rev. 2015, 44, 4501. 25652670 10.1039/c4cs00345d

[123] F. Chen , L. Teng , C. Lu , C. Zhang , Q. Rong , Y. Zhao , Y. Yang , Y. Wang , G. Song , X. Zhang , Anal. Chem. 2020, 92, 13452. 32900179 10.1021/acs.analchem.0c02859

[124] C. Sun , J. S. H. Lee , M. Zhang , Adv. Drug Delivery Rev. 2008, 60, 1252. 10.1016/j.addr.2008.03.018 PMC2702670 18558452

[125] D. Ghosh , Y. Lee , S. Thomas , A. G. Kohli , D. S. Yun , A. M. Belcher , K. A. Kelly , Nat. Nanotechnol. 2012, 7, 677. 22983492 10.1038/nnano.2012.146 PMC4059198

[126] J. Shin , R. M. Anisur , M. K. Ko , G. H. Im , J. H. Lee , I. S. Lee , Angew. Chem., Int. Ed. 2009, 48, 321. 10.1002/anie.200802323 19040234

[127] J. L. Bridot , A. C. Faure , S. Laurent , C. Rivie`re , C. Billotey , B. Hiba , M. Janier , V. Josserand , J. L. Coll , L. V. Elst , R. Muller , S. Roux , P. Perriat , O. Tillement , J. Am. Chem. Soc. 2007, 129, 5076. 17397154 10.1021/ja068356j

[128] R. Yue , C. Zhang , L. Xu , Y. Wang , G. Guan , L. Lei , X. Zhang , G. Song , Chem. 2022, 8, 1956.

[129] Y. Kuang , Y. Zhang , Y. Zhao , Y. Cao , Y. Zhang , Y. Chong , R. Pei , ACS Appl. Mater. Interfaces 2020, 12, 35928. 32686939 10.1021/acsami.0c09422

[130] Y. Liu , Y. Tang , Y. Tian , J. Wu , J. Sun , Z. Teng , S. Wang , G. Lu , ACS Appl. Nano Mater. 2019, 2, 1194.

[131] K. He , J. Li , Y. Shen , Y. Yu , J. Mater. Chem. B 2019, 7, 6840. 31609370 10.1039/c9tb01654f

[132] E. Terreno , D. D. Castelli , A. Viale , S. Aime , Chem. Rev. 2010, 110, 3019. 20415475 10.1021/cr100025t

[133] B. Y. Hsu , M. Ng , A. Tan , J. Connell , T. Roberts , M. Lythgoe , Y. Zhang , S. Y. Wong , K. Bhakoo , A. M. Seifalian , X. Li , J. Wang , Adv. Healthcare Mater. 2016, 5, 721. 10.1002/adhm.201500908 26895111

[134] T. Kim , E. J. Cho , Y. Chae , M. Kim , A. Oh , J. Jin , E. S. Lee , H. Baik , S. Haam , J. S. Suh , Y. M. Huh , K. Lee , Angew. Chem., Int. Ed. 2011, 50, 10589. 10.1002/anie.201103108 21928456

[135] C. Dai , H. Lin , G. Xu , Z. Liu , R. Wu , Y. Chen , Chem. Mater. 2017, 29, 8637.

[136] B. H. Kim , N. Lee , H. Kim , K. An , Y. I. Park , Y. Choi , K. Shin , Y. Lee , S. G. Kwon , H. B. Na , J. G. Park , T. Y. Ahn , Y. W. Kim , W. K. Moon , S. H. Choi , T. Hyeon , J. Am. Chem. Soc. 2011, 133, 12624. 21744804 10.1021/ja203340u

[137] D. Ling , W. Park , S. J. Park , Y. Lu , K. S. Kim , M. J. Hackett , B. H. Kim , H. Yim , Y. S. Jeon , K. Na , T. Hyeon , J. Am. Chem. Soc. 2014, 136, 5647. 24689550 10.1021/ja4108287

[138] Y. W. Jun , J. H. Lee , J. Cheon , Angew. Chem., Int. Ed. 2008, 47, 5122. 10.1002/anie.200701674 18574805

[139] S. A. Corr , S. J. Byrne , R. Tekoriute , C. J. Meledandri , D. F. Brougham , M. Lynch , C. Kerskens , L. O'Dwyer , Y. K. Gun'ko , J. Am. Chem. Soc. 2008, 130, 4214. 18331033 10.1021/ja710172z

[140] Y. G., Lee , W. P. Qian , L. Wang , Y. A. Wang , C. A. Staley , M. Satpathy , S. Nie , H. Mao , L. Yang , ACS Nano 2013, 7, 2078. 23402593 10.1021/nn3043463 PMC3609912

[141] Z. Shen , H. Wu , S. Yang , X. Ma , Z. Li , M. Tan , A. Wu , Biomaterials 2015, 70, 1. 26295434 10.1016/j.biomaterials.2015.08.022

[142] Y. Huang , K. Mao , B. Zhang , Y. Zhao , Mater. Sci. Eng., C 2017, 70, 763. 10.1016/j.msec.2016.09.052 27770953

[143] H. Tang , Y. Guo , L. Peng , H. Fang , Z. Wang , Y. Zheng , H. Ran , Y. Chen , ACS Appl. Mater. Interfaces 2018, 10, 15428. 29652130 10.1021/acsami.8b01967

[144] S. Yang , D. Chen , N. Li , X. Mei , X. Qi , H. Li , Q. Xu , J. Lu , J. Mater. Chem. 2012, 22, 25354.

[145] L. Jiang , Q. Zhou , K. Mu , H. Xie , Y. Zhu , W. Zhu , Y. Zhao , H. Xu , X. Yang , Biomaterials 2013, 34, 7418. 23810255 10.1016/j.biomaterials.2013.05.078

[146] Z. Xiao , L. Chan , D. Zhang , C. Huang , C. Mei , P. Gao , Y. Huang , J. Liang , L. He , C. Shi , T. Chen , L. Luo , J. Mater. Chem. B 2019, 7, 2926.

[147] Y. Wang , R. Huang , G. Liang , Z. Zhang , P. Zhang , S. Yu , J. Kong , Small 2014, 10, 109. 23828738 10.1002/smll.201301297

[148] M. Cai , B. Li , L. Lin , J. Huang , Y. An , W. Huang , Z. Zhou , Y. Wang , X. Shuai , K. Zhu , Biomater. Sci. 2020, 8, 3485. 32432234 10.1039/d0bm00295j

[149] P. Zou , Y. K. Yu , Y. A. Wang , Y. Q. Zhong , A. Welton , C. Galba´n , S. M. Wang , D. X. Sun , Mol. Pharm. 2010, 7, 1974. 20845930 10.1021/mp100273t PMC2997864

[150] C. Lin , B. Chi , C. Xu , C. Zhang , F. Tian , Z. Xu , L. Li , A. K. Whittaker , J. Wang , J. Mater. Chem. B 2019, 7, 6612. 31591619 10.1039/c9tb01509d

[151] C. Lu , C. Zhang , P. Wang , Y. Zhao , Y. Yang , Y. Wang , H. Yuan , S. Qu , X. Zhang , G. Song , K. Pu , Chem. 2020, 6, 2314.

[152] L. Jin , J. Liu , Y. Tang , L. Cao , T. Zhang , Q. Yuan , Y. Wang , H. Zhang , ACS Appl. Mater. Interfaces 2017, 9, 41648. 29116748 10.1021/acsami.7b10608

[153] H. B. Na , J. H. Lee , K. An , Y. I. Park , M. Park , I. S. Lee , D. H. Nam , S. T. Kim , S. H. Kim , S. W. Kim , K. H. Lim , K. S. Kim , S. O. Kim , T. Hyeon , Angew. Chem., Int. Ed. 2007, 46, 5397. 10.1002/anie.200604775 17357103

[154] W. S. Seo , J. H. Lee , X. Sun , Y. Suzuki , D. Mann , Z. Liu , M. Terashima , P. C. Yang , M. V. McConnell , D. G. Nishimura , H. Dai , Nat. Mater. 2006, 5, 971. 17115025 10.1038/nmat1775

[155] G. Zhang , R. Du , L. Zhang , D. Cai , X. Sun , Y. Zhou , J. Zhou , J. Qian , K. Zhong , K. Zheng , D. Kaigler , W. Liu , X. Zhang , D. Zou , Z. Wu , Adv. Funct. Mater. 2015, 25, 6101.

[156] X. Sun , R. Du , L. Zhang , G. Zhang , X. Zheng , J. Qian , X. Tian , J. Zhou , J. He , Y. Wang , Y. Wu , K. Zhong , D. Cai , D. Zou , Z. Wu , ACS Nano 2017, 11, 7049. 28665575 10.1021/acsnano.7b02675

[157] X. Huang , Y. Yuan , W. Ruan , L. Liu , M. Liu , S. Chen , X. Zhou , J. Nanobiotechnol. 2018, 16, 30. 10.1186/s12951-018-0350-5 PMC5870481 29587764

[158] X. Jing , Y. Xu , D. Liu , Y. Wu , N. Zhou , D. Wang , K. Yan , L. Meng , Nanoscale 2019, 11, 15508. 31393496 10.1039/c9nr04768a

[159] G. Guan , C. Zhang , H. Liu , Y. Wang , Z. Dong , C. Lu , B. Nan , R. Yue , X. Yin , X. B. Zhang , G. Song , Angew. Chem., Int. Ed. 2022, 61, e202117229. 10.1002/anie.202117229 35460321

[160] B. A. Chabner , T. G. Roberts , Nat. Rev. Cancer 2005, 5, 65. 15630416 10.1038/nrc1529

[161] L. Jia , X. Li , H. Liu , J. Xia , X. Shi , M. Shen , Nano Today 2021, 36, 101022.

[162] H. Gao , X. Liu , W. Tang , D. Niu , B. Zhou , H. Zhang , W. Liu , B. Gu , X. Zhou , Y. Zheng , Y. Sun , X. Jia , L. Zhou , Nanoscale 2016, 8, 19573. 27874119 10.1039/c6nr07062k

[163] Z. Jiang , B. Yuan , N. Qiu , Y. Wang , L. Sun , Z. Wei , Y. Li , J. Zheng , Y. Jin , Y. Li , S. Du , J. Li , A. Wu , Nano‐Micro Lett. 2019, 11, 61. 10.1007/s40820-019-0292-y PMC7770799 34138009

[164] Y. Liu , L. Feng , T. Liu , L. Zhang , Y. Yao , D. Yu , L. Wang , N. Zhang , Nanoscale 2014, 6, 3231. 24500240 10.1039/c3nr05647c

[165] B. Li , M. Cai , L. Lin , W. Sun , Z. Zhou , S. Wang , Y. Wang , K. Zhu , X. Shuai , Biomater. Sci. 2019, 7, 1529. 30681081 10.1039/c8bm01501e

[166] X. Zhao , Y. Qiu , Y. Miao , Z. Liu , W. Yang , H. Hou , ACS Appl. Nano Mater. 2018, 1, 2621.

[167] Y. Hao , L. Wang , B. Zhang , D. Li , D. Meng , J. Shi , H. Zhang , Z. Zhang , Y. Zhang , Int. J. Nanomed. 2016, 11, 1759. 10.2147/IJN.S98832 PMC4857809 27199556

[168] D. X. Ye , Y. Y. Ma , W. Zhao , H. M. Cao , J. L. Kong , H. M. Xiong , H. Mohwald , ACS Nano 2016, 10, 4294. 27018822 10.1021/acsnano.5b07846

[169] M. O. Abdalla , P. Karna , H. K. Sajja , H. Mao , C. Yates , T. Turner , R. Aneja , J. Controlled Release 2011, 149, 314. 10.1016/j.jconrel.2010.10.030 PMC3179860 21047537

[170] J. Wang , X. Wang , Y. Song , J. Wang , C. Zhang , C. Chang , J. Yan , L. Qiu , M. Wu , Z. Guo , Chem. Sci. 2013, 4, 2605.

[171] L. Wu , M. Wu , X. Lin , X. Zhang , X. Liu , J. Liu , J. Mater. Chem. B 2017, 5, 849. 32263853 10.1039/c6tb02804g

[172] M. X. Wu , J. Gao , F. Wang , J. Yang , N. Song , X. Jin , P. Mi , J. Tian , J. Luo , F. Liang , Y. W. Yang , Small 2018, 14, e1704440. 29611291 10.1002/smll.201704440

[173] B. Dutta , A. Nema , N. G. Shetake , J. Gupta , K. C. Barick , M. A. Lawande , B. N. Pandey , I. K. Priyadarsini , P. A. Hassan , Mater. Sci. Eng., C 2020, 112, 110915. 10.1016/j.msec.2020.110915 32409067

[174] L. Li , C. Zhang , R. Zhang , Z. Xu , Z. Xu , A. K. Whittaker , ACS Appl. Nano Mater. 2018, 1, 5027.

[175] W. Zhu , Z. Dong , T. Fu , J. Liu , Q. Chen , Y. Li , R. Zhu , L. Xu , Z. Liu , Adv. Funct. Mater. 2016, 26, 5490.

[176] S. Chen , Q. Jia , X. Zheng , Y. Wen , W. Liu , H. Zhang , J. Ge , P. Wang , Sci. China Mater. 2018, 61, 1325.

[177] Z. Zha , X. Yue , Q. Ren , Z. Dai , Adv. Mater. 2013, 25, 777. 23143782 10.1002/adma.201202211

[178] G. Song , M. Kenney , Y.‐S. Chen , X. Zheng , Y. Deng , Z. Chen , S. X. Wang , S. S. Gambhir , H. Dai , J. Rao , Nat. Biomed. Eng. 2020, 4, 325. 32015409 10.1038/s41551-019-0506-0 PMC7071985

[179] B. Yin , Y. Wang , C. Zhang , Y. Zhao , Y. Wang , L. Teng , Y. Yang , Z. Zeng , S. Huan , G. Song , X. Zhang , Anal. Chem. 2019, 91, 15275. 31674180 10.1021/acs.analchem.9b04429

[180] Y. Wang , L. Shi , Z. Ye , K. Guan , L. Teng , J. Wu , X. Yin , G. Song , X. B. Zhang , Nano Lett. 2020, 20, 176. 31777250 10.1021/acs.nanolett.9b03556

[181] L. Shi , Y. Wang , C. Zhang , Y. Zhao , C. Lu , B. Yin , Y. Yang , X. Gong , L. Teng , Y. Liu , X. Zhang , G. Song , Angew. Chem., Int. Ed. 2021, 60, 9562. 10.1002/anie.202014415 33590957

[182] S. A. Rosenberg , J. C. Yang , N. P. Restifo , Nat. Med. 2004, 10, 909. 15340416 10.1038/nm1100 PMC1435696

[183] X. Li , X. Wang , A. Ito , Chem. Soc. Rev. 2018, 47, 4954. 29911725 10.1039/c8cs00028j

[184] W. Nie , W. Wei , L. Zuo , C. Lv , F. Zhang , G. H. Lu , F. Li , G. Wu , L. L. Huang , X. Xi , H. Y. Xie , ACS Nano 2019, 13, 1469. 30763076 10.1021/acsnano.8b07141

[185] R. Ge , M. Lin , X. Li , S. Liu , W. Wang , S. Li , X. Zhang , Y. Liu , L. Liu , F. Shi , H. Sun , H. Zhang , B. Yang , ACS Appl. Mater. Interfaces 2017, 9, 19706. 28553876 10.1021/acsami.7b05583

[186] G. Shu , M. Chen , J. Song , X. Xu , C. Lu , Y. Du , M. Xu , Z. Zhao , M. Zhu , K. Fan , X. Fan , S. Fang , B. Tang , Y. Dai , Y. Du , J. Ji , Bioact. Mater. 2021, 6, 1423. 33210034 10.1016/j.bioactmat.2020.10.020 PMC7658445

[187] M. Cao , P. Wang , Y. Kou , J. Wang , J. Liu , Y. Li , J. Li , L. Wang , C. Chen , ACS Appl. Mater. Interfaces 2015, 7, 25014. 26418578 10.1021/acsami.5b06938

[188] C. Wang , L. Zhang , S. Li , M. Zhang , T. Wang , L. Li , C. Wang , Z. Su , J. Mater. Chem. B 2016, 4, 5809. 32263753 10.1039/c6tb01669c

[189] X. Lin , X. Song , Y. Zhang , Y. Cao , Y. Xue , F. Wu , F. Yu , M. Wu , X. Zhu , Biomater. Sci. 2020, 8, 1875. 32010912 10.1039/c9bm01482a

[190] D. Zhang , Z. Cai , N. Liao , S. Lan , M. Wu , H. Sun , Z. Wei , J. Li , X. Liu , Chem. Sci. 2018, 9, 7390. 30542542 10.1039/c8sc02408a PMC6237124

[191] O. Tredan , A. B. Garbens , A. S. Lalani , I. F. Tannock , Cancer Res. 2009, 69, 940. 19176397 10.1158/0008-5472.CAN-08-0676

[192] F. Benyettou , G. Das , A. R. Nair , T. Prakasam , D. B. Shinde , S. K. Sharma , J. Whelan , Y. Lalatonne , H. Traboulsi , R. Pasricha , O. Abdullah , R. Jagannathan , Z. Lai , L. Motte , F. Gandara , K. C. Sadler , A. Trabolsi , J. Am. Chem. Soc. 2020, 142, 18782. 33090806 10.1021/jacs.0c05381

[193] J. Liu , Q. Chen , W. Zhu , X. Yi , Y. Yang , Z. Dong , Z. Liu , Adv. Funct. Mater. 2017, 27, 1605926.

[194] L. Feng , L. Cheng , Z. Dong , D. Tao , T. E. Barnhart , W. Cai , M. Chen , Z. Liu , ACS Nano 2017, 11, 927. 28027442 10.1021/acsnano.6b07525 PMC5372701

[195] J. Xiao , G. Zhang , R. Xu , H. Chen , H. Wang , G. Tian , B. Wang , C. Yang , G. Bai , Z. Zhang , H. Yang , K. Zhong , D. Zou , Z. Wu , Biomaterials 2019, 216, 119254. 31195303 10.1016/j.biomaterials.2019.119254

[196] M. Li , Q. Zhao , X. Yi , X. Zhong , G. Song , Z. Chai , Z. Liu , K. Yang , ACS Appl. Mater. Interfaces 2016, 8, 9557. 27039932 10.1021/acsami.5b11588

[197] F. Su , X. Zhao , C. Dai , Y. Li , C. Yang , X. Yan , ChemComm 2019, 55, 5283. 10.1039/c9cc01446b 30993283

[198] M. Song , N. Liu , L. He , G. Liu , D. Ling , X. Su , X. Sun , Nano Res. 2018, 11, 2796.

[199] J. Wahsner , E. M. Gale , A. Rodriguez‐Rodriguez , P. Caravan , Chem. Rev. 2019, 119, 957. 30350585 10.1021/acs.chemrev.8b00363 PMC6516866

